# ﻿Morphology, taxonomy, biogeography and ecology of *Micrasteriasfoliacea* Bailey ex Ralfs (Desmidiales, Zygnematophyceae)

**DOI:** 10.3897/phytokeys.226.103500

**Published:** 2023-05-09

**Authors:** Anatoliy Levanets, Sanet Janse van Vuuren

**Affiliations:** 1 Unit for Environmental Sciences and Management, North-West University, Private Bag X6001, Potchefstroom, 2520, South Africa North-West University Potchefstroom South Africa

**Keywords:** Botswana, global distribution, Mozambique, new records, taxonomy

## Abstract

*Micrasteriasfoliacea* (Desmidiales, Zygnematophyceae) is an interesting desmid species as its filamentous life form is quite different from all other species within the genus. Due to the large size of the filaments and cells, accurate species identification is easy. After its original description from Rhode Island (USA) it was recorded from five continents, but no record could be found of its presence in Europe. In this paper a review of the worldwide distribution of *M.foliacea* (Desmidiales, Zygnematophyceae) is presented, together with notes on the species’ ecology. In addition to its currently known geographical distribution, the paper also records the species’ presence at two new locations in southern Africa, namely Botswana (Okavango River) and Mozambique (Palma, Cabo Delgado). The paper presents a discussion of taxonomical levels of intraspecific taxa, based on morphological characteristics. It is proposed that the taxonomical status of M.foliaceaBailey ex Ralfsf.nodosa should be raised to the variety, as its nodular cell wall thickenings are unique morphological features.

## ﻿Introduction

The genus *Micrasterias* C. Agardh ex Ralfs (1848: 48) (Desmidiales, Zygnematophyceae) accounts for 95 currently taxonomically accepted species (Guiry and Guiry 2023), however in AlgaeBase there are more than 900 species and intraspecific names (Guiry and Guiry 2023).

Species of the genus *Micrasterias* consists mostly of single cells, each divided into two symmetrical semicells which are mirror images of each other. There is only one filamentous species within the genus, namely *Micrasteriasfoliacea* Bailey ex Ralfs. This species was initially described from Rhode Island (United States of America) in a letter from Prof. J.W. Bailey addressed to John Ralfs in 1847, and it was published and illustrated by the latter in the British Desmidieae (Ralfs 1848: 210).

At later stages *M.foliacea* was also recorded in Asia (Bangladesh, Cambodia, China, India, Indonesia, Japan, Malaysia, Myanmar, Nepal, Papua New Guinea, Pakistan, Philippines, Russia, Singapore, South Korea, Sri Lanka, Taiwan, Thailand, and Vietnam), North America (Canada, USA), Central America (Cuba, Nicaragua, Panama) and South America (Argentina, Bolivia, Brazil, Suriname, Venezuela), Australia and Africa. Currently, this species is known from 17 countries on the African continent, mostly from tropical central Africa (Benin, Cameroon, Chad, Côte d’Ivoire, Democratic Republic of Congo, Guinea, Mali, Niger, Nigeria, Sierra Leone, Tanzania), Madagascar, and southern Africa (Botswana, Mozambique, South Africa, Zambia, Zimbabwe).

In addition to the type species, several forms and varieties were described from tropical regions of Asia and two from South America based on cell dimensions and morphology (ornamentation, size and position of the spines, cell wall thickenings).

In this paper we report on the presence of this species in two new locations in southern Africa, namely the Okavango River in Botswana and a wetland in northern Mozambique.

## ﻿Material and methods

In Botswana grab samples were collected in the Okavango River near Shakawe during January 2015. The samples were fixed with 10% ethanol to preserve the algae. The water lily, genus *Nymphaea* L., dominated at the sampling site during sampling.

In Mozambique samples were collected during October 2011 near Palma, Cabo Delgado, just south of the Tanzanian border. Water samples were collected from seven sites in an inland wetland system with soft black peat-like sediment. All samples were preserved with 10% ethanol. The samples in which *M.foliacea* were found were deposited in the North-West University Diatom Collection and Herbarium (sample no. 12-140, 12-142 and 12-436).

The samples were examined using a Leica DM2500 LED compound microscope equipped with phase contrast objectives and a Flexacam C3 microscope digital camera.

An investigation of all scientific literature, phycological inventories, technical reports and internet databases (Atlas of Living Australia 2022; Guiry and Guiry 2023; The Global Biodiversity Information Facility 2023) was carried out to account for all existing records of *M.foliacea*, including intraspecific names and nomenclatural synonyms. Where possible, the citation, along with geographical locations and ecological characteristics of the habitats, was recorded. A desktop study of all published scientific literature, mentioning the ecology of the species, was also made to determine its ecological preferences.

## ﻿Results and discussion

In the following paragraphs the taxonomy, morphology, geographical distribution, habitat and ecology of the different varieties of *M.foliacea* will be discussed.

### ﻿1. Micrasteriasfoliacea[var.foliacea]

Taxon classificationPlantaeDesmidialesDesmidiaceae

 Bailey ex Ralfs, 1848

835C972E-F12B-5487-9499-0602066C39FF

Micrasterias foliacea [var. foliacea] Bailey ex Ralfs, 1848. “The British Desmidiaceae”: 210, tab. 35, fig. 3.

#### Synonyms.

*M.foliacea* Bailey *in lit. cum icone* 1847; *M.foliacea* Bailey ex Ralfs f. α typica Turner, 1892. “Algae Aquae Dulcis Indiae Orientalis”: 94, tab. 6, figs 12–14; M.foliaceaBailey ex Ralfsvar.granulifera J.A. Cushman, 1908. “Rhodora” 10(114): 111.

The earliest description of M.foliaceavar.granulifera by Cushman (1908) is doubtful, and no drawings or micrographs were provided. Cushman (1908) indicated that var. granulifera is similar to the type, but in addition the surface is covered with large irregularly disposed granules. Later, Krieger (1939) classified this variety as a synonym of the type variety, which is currently accepted.

#### Morphology.

**Figure 1.**
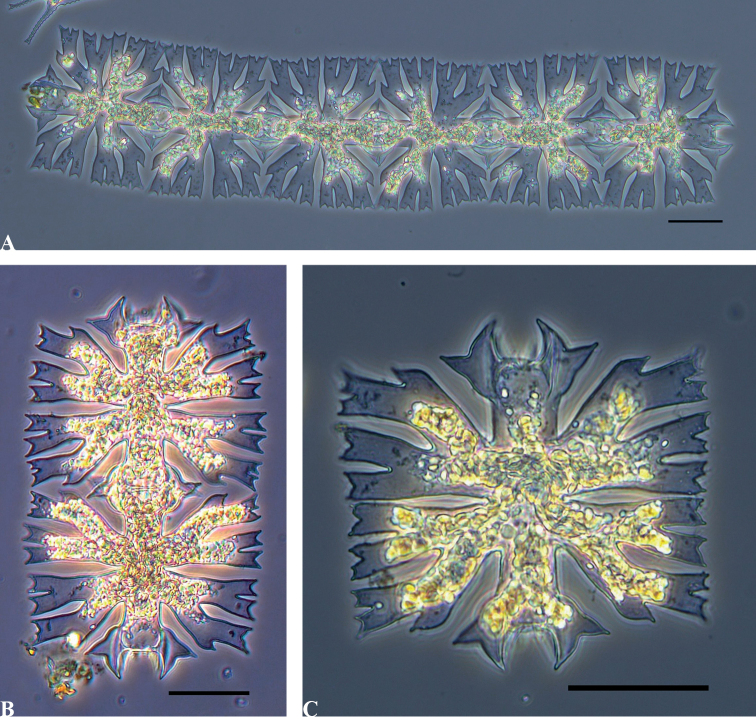
Morphology of M.foliaceavar.foliacea found in samples from Mozambique **A** six cells illustrating chain formation **B** two adjacent cells indicating the interlocking polar extensions **C** morphology of a single cell. Scale bars: 30 µm.

Fig. 1 illustrates the morphology of M.foliaceavar.foliacea found in the Mozambique samples. It is the only species of *Micrasterias* where the cells are permanently attached to each other to form a ribbon-like chain, which may consist of 2–100 cells. The cells are nearly square (sub-quadrate) with deep constrictions at the sinus. Each semicell is sub-divided into three lobes, two of which are lateral and the third apical (polar). The lateral lobes are further sub-divided into smaller lobes and lobules by means of incisions of various depths. The polar lobes are narrow with a pair of pointed extensions at each end. The polar extensions interlock where neighbouring cells are attached to each other. The cells are 70–80 µm in length and 65–80 µm wide (isthmus 13–16 µm). The morphology of specimens in our samples corresponds to the original description by Ralfs (1848) and later descriptions, such as those provided by Kim (2013) and Ribeiro et al. (2015).

A description of Ling and Taylor (2000) of a form of *M.foliacea* recorded from the Northern Territories in Australia indicated a simpler form with less divided lateral lobes and one, instead of the usual two, triangular process on the face of the polar lobe. Cell length without processes was 66–67 μm, and with processes 91–95 μm; width was 102–109 μm; apex 38–43 μm and isthmus 14–15 μm.

#### Distribution.

**Figure 2.**
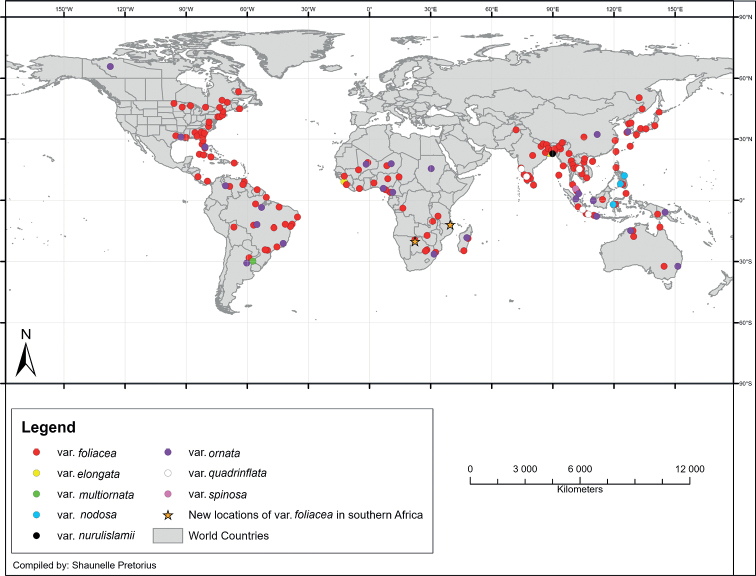
Worldwide distribution of different varieties of *Micrasteriasfoliacea*.

A map, illustrating the geographical distribution of *M.foliacea*, is presented in Fig. 2. This figure shows that variety *foliacea* (type) is widely distributed, being present on most continents. It is, however, only recorded from the eastern side of North America and, surprisingly, it is completely absent from European countries. Palamar-Mordvintseva (1984) speculated that this species can possibly be found in future in the rice fields of southern Ukraine, but according to the general known distribution of this species it is doubtful that this statement is true. A detailed list, with references, of all localities where this variety was found is presented in Suppl. material 1.

#### Habitat and ecology.

From intensive literature searches it is clear that the habitats of free-living *M.foliacea* are exceptionally diverse. It can be found in plankton and periphyton of a variety of different lentic and lotic water bodies. Lentic (stagnant) water bodies include wetlands, marshes, various types of swamps (e.g., peat swamps) and ponds (including irrigation and oxidation ponds, rainwater ponds, rock ponds, fishponds, and small ponds in botanical gardens), reservoirs, deltas, floodplains, lagoons and lakes (including crater lakes), flooded savannahs and meadows, and in ditches associated with paddy (rice) fields. It is most common in small and shallow (<1 to 2 m) ponds, but it was also occasionally found at the embankments of larger standing water bodies, e.g., Lake Laguna de Bay in the Philippines (4–6 m deep; Behre 1956). Lotic habitats include cobbles, streams, rivers, and irrigation canals. In addition, it was also recorded from a tropical estuarine mangrove swamp in Nigeria with high conductivity levels (Table 1; Ubong et al. 2017). In these habitats, it often occurs between floating algal masses. In terrestrial habitats it may be found amongst moist mosses, growing on the surface of rocks, or inhabiting wet soils.

**Table 1.** Physico-chemical properties of waterbodies throughout the world in which M.foliaceavar.foliacea was found.

Environmental variable	Value/Ranges	Country	Reference
pH	6.6	Cambodia	Hirano 1972
	4.7–5.3	Malaysia	Ratnasabapathy and Kumano 1974
	5.78±0.76, 6.10±0.44, 6.12±0.74	Malaysia	Noor et al. 2012
	8.0	Philippines	Behre 1956
	5.0–5.5	Sumatra	Scott and Prescott 1961
	6.3–6.8	Russia	Gontcharov 1997
	5.6–7.5	South Korea	Kim 2014
	5.6, 5.9	Thailand	Ngearnpat et al. 2008
	7.42±0.11	Thailand	Prasertsin and Peerepornpisal 2018
	7.6–8.3	India	Ecology and biodiversity of Lower Ganga River basin 2012
	6.5–7.0	India	Kalita et al. 2016,
	5.7–6.8 (open water), 5.2 (littoral vegetation)	Zambia	Thomasson 1966
	6.3	Congo	Van Oye 1953
	8.0	Nigeria	Ubong et al. 2017
	6.2	South Africa	Claassen 1982
	8.15	Nigeria	Ali et al. 2016
	6.0	India	Das and Keshri 2013, 2016
	6.44	Botswana	Williamson and Marazzi 2013
	5.7, 8.1	USA	Oyadomari 2013
	5.4	Brazil	Thomasson 1977
	5.5	Suriname	Leentvaar 1975
	5.26–5.83	Australia	Thomasson 1986
	6.8–7.2	USA	Ngo et al. 1986–1987
Water temperature (°C)	26.8	Cambodia	Hirano 1972
	23.8–26.9 (running water) 23.4–33.4 (still water)	Malaysia	Ratnasabapathy and Kumano 1974
	27.01±4.2, 27.22±3.96, 27.33±4.87	Malaysia	Noor et al. 2012
	8, 10, 21	India	Das and Keshri 2016
	22–26	Russia	Gontcharov 1997
	29.1, 32.2	Thailand	Ngearnpat et al. 2008
	30±0.71	Thailand	Prasertsin and Peerepornpisal 2018
	18.6–30.4	India	Ecology and biodiversity of Lower Ganga River basin 2012
	17.8–30.2	India	Kalita et al. 2016
	35	Guinea	Bourrelly 1975
	23–24	USA	Oyadomari 2013
	28.5–31.1	Australia	Thomasson 1986
	10	India	Das and Keshri 2013
	21.6	Botswana	Williamson and Marazzi 2013
	33.46	Nigeria	Ali et al. 2016
	26.17	Nigeria	Ubong et al. 2017
Conductivity (μS/cm)	31.88±17, 33.88±12, 34.75±19	Malaysia	Noor et al. 2012
	36–55, 44–108	South Korea	Kim 2014
	315, 327	Thailand	Ngearnpat et al. 2008
	15, 6	USA	Oyadomari 2013
	23–58	Australia	Thomasson 1986
	76.0	Botswana	Williamson and Marazzi 2013
	74.34	Nigeria	Ali et al. 2016
	49993.33 ± 634.09	Nigeria	Ubong et al. 2017
	94.80±2.39	Thailand	Prasertsin and Peerepornpisal 2018
Dissolved oxygen (mg/L)	2.35±2.62, 3.16±2.22, 3.44±2.76	Malaysia	Noor et al. 2012
	4.2, 8.0	Thailand	Ngearnpat et al. 2008
	3.69–6.67	Australia	Thomasson 1986
	1.70	Botswana	Williamson and Marazzi 2013
	5.74	Nigeria	Ali et al. 2016
	6.12	Nigeria	Ubong et al. 2017
	7.10±0.79	Thailand	Prasertsin and Peerepornpisal 2018
Alkalinity (meq/L)	0.038–0.082	Australia	Thomasson 1986
	2.53	Nigeria	Ali et al. 2016
	149.00±2.65	Thailand	Prasertsin and Peerepornpisal 2018
Total alkalinity (mg/L)	11, 11.5	Thailand	Ngearnpat et al. 2008
Phosphate-phosphorus (mg/L)	0.13±0.28 0.15±0.39 0.18±0.45	Malaysia	Noor et al. 2012
	0.66	Nigeria	Ali et al. 2016
Soluble Reactive Phosphate (mg/L)	0.52±0.28	Thailand	Prasertsin and Peerepornpisal 2018
Nitrate-nitrogen (mg/L)	0.15±0.1 0.15±0.2 0.18±0.2	Malaysia	Noor et al. 2012
	12.63	Nigeria	Ali et al. 2016
Turbidity (NTU)	1.6	Botswana	Williamson and Marazzi 2013
	0.46	Nigeria	Ali et al. 2016
	25.55	Nigeria	Ubong et al. 2017
	8.07±1.7	Thailand	Prasertsin and Peerepornpisal 2018

Besides being free-living, it can also be found as epiphytes on aquatic plants (Jena and Adhikary 2011). It is frequently found living on *Utricularia* species, such as *U.flexuosa* (Lütkemüller 1900; Turner 1892) and *U.fasciculata* (Turner 1892). It was also found on leaves of *Hymenachneamplexicaulis* (Salazár Pereira 1991; Salazar 2006/2007) and the stems and roots of submerged *Ipomaeareptans* (Bourrelly 1975). It was common in samples that were obtained when the roots of *Eichhorniacrassipes*, *Pistiastratiotes*, *Salviniamolesta* and submerged aquatic macrophytes were squeezed out (Bicudo and Sormus 1982; Rai et al. 2008; Paudel 2017). The species was also found in the stomachs of catfish *Synodontisschall* and *S.nigrita* (Lalèyè et al. 2006).

A variety of aquatic plants inhabit waters in which M.foliaceavar.foliacea was found. Shallow water was often dominated by species of *Nymphaea* and *Utricularia* (Schumacher 1956; Thomasson 1966; Ratnasabapathy and Kumano 1974; Turner 1892; González 2009). Other dominant species include those of *Pistia* and *Salvinia* (Ngearnpat et al. 2008; González 2009). In deeper waters *Braseniaschreberi*, *Cabombacaroliniana*, *Nupharorbiculata*, *Nymphaeaodorata* and *Utriculariapurpurea* were common (Schumacher 1956). During the current study *Nymphaea* dominated the macrophyte community at the sampling site in Botswana.

The abundance of M.foliaceavar.foliacea may range from extremely rare, present in low to moderate quantities (Behre 1956) or it may be common (Scott and Prescott 1961; Prowse 1969; Kadiri and Opute 1989; Kadiri 2002; Islam and Ifranullah 2006). Other authors described it as abundant (Kim 2014), or sometimes dominant (Medvedeva 2007; Ekhator et al. 2013).

Variety *foliacea* can be found in a wide pH range and a literature overview, presented in Table 1, shows that it is present in both acidic (min pH 4.7; Malaysia), as well as alkaline waters (max pH 8.3; India). Results from the literature review contradict a finding made by Prescott and Scott (1943) who stated that the acidity of water in which *M.foliacea* grows, varies between pH 5.8 and 6.4 (rarely as high as 6.8), and that this species’ distribution is so specifically related to the chemistry of the water that it can be used as an indicator organism for soft and highly acid waters. After the study of Prescott and Scott (1943) the species was frequently recorded in waters with pH values around 8 (Philippines, India, Nigeria, USA; see Table 1), indicating that it can also tolerate and thrive in alkaline waters and that it is thus not suitable as an indicator organism. Table 1 also indicates alkalinity ranges of water in which the species was recorded and, in accordance with the findings of Förster (1982), several published results show that it is usually found under neutral to alkaline conditions.

It was found in both winter (Islam and Ifranullah 2006) and summer (Thomas et al. 2003; Prasertsin and Peerapornpisal 2018) seasons throughout the world. In India it was found during pre-monsoon, monsoon, post-monsoon and winter seasons (Kalita et al. 2016). Water temperature ranged between a minimum of 8 °C (India) and a maximum of 35 °C (Guinea), although it was most frequently recorded in moderate to high water temperatures, ranging from 23–27 °C (Table 1).

Typical conductivity levels of freshwater are below 1 500 µS/cm, while typical sea water has a conductivity value of about 50 000 µS/cm. *M.foliacea* was usually found in freshwater with relatively low conductivity values (< 327 µS/cm; Table 1), but cells were also found in some estuaries, archipelagos and marine waters (Chung et al. 1965; McAlice 1975; Opute 1992; Kadiri 2002; Li et al. 2011; Ecology and biodiversity of Lower Ganga River basin 2012; Silveira 2012; Opute and Kadiri 2013; Dayala et al. 2014; Mama et al. 2016; Ubong et al. 2017). Average conductivity values in the tropical estuarine mangrove swamp (Nigeria) in which it was found by Ubong et al. (2017) was in the order of 50 000 μS/cm, showing its ability to survive in saline water. It is, however, suspected that its presence in estuaries and oceans may be the result of outflows from the rivers.

Table 1 shows nutrient ranges (phosphate-phosphorus, nitrate-nitrogen) of water in which *M.foliacea* was recorded. Based on these nutrient concentrations, it was mostly found in oligotrophic to slightly mesotrophic water (Kim 2014; Ali et al. 2016).

Dissolved oxygen concentrations ranged from 1.7–8.0 mg/L in water in which *M.foliacea* was recorded. It was mostly found in low turbidity (high transparency) waters (Williamson and Marazzi 2013; Ali et al. 2016; Ubong et al. 2017; Prasertsin and Peerapornpisal 2018). However, in Thailand it was found in muddy, pale yellow-brown water with transparencies less than 1 m (Hirano 1975) and in the middle reaches of the Niger River in Mali it was found in polluted, cloudy, muddy water having a sandy substrate (Couté and Rousselin 1975).

### ﻿2. Micrasterias foliacea var. elongata

Taxon classificationPlantaeDesmidialesDesmidiaceae

(W.B. Turner) Willi Krieger, 1939

F4D31970-82D2-5773-B0B6-B2AAA68705D3

Micrasterias foliacea var. elongata (W.B. Turner) Willi Krieger, 1939. “Rabenhorst’s Kryptogamen-Flora von Deutschland”, “Österreich und der Schweiz”, 2 Aufl., 13 (Abt.1, Teil 2): 77.

#### Synonyms.

*M.foliacea* Bailey ex Ralfs var. β Wallich, 1860. “Annals and Magazine of Natural History” Series 3, 5: 280, tab. XIV, figs 1–4; M.foliaceaBailey ex Ralfsf. βelongata Turner, 1892. “Aquae Dulcis Indiae Orientalis”: 94.

This variety was originally described by Wallich (1860) as “var. β”. A later study by Turner (1892) revealed the same alga, but he described it as “forma β *elongata*”. In his monograph, Krieger (1939) indicated the same algal species as “var. elongata Turner”.

#### Morphology.

The prominent feature of M.foliaceavar.elongata is the largely developed, triangular terminal lobe, with an outward directed base, emarginate at its angles and centre and furnished with two short stout teeth placed obliquely to each other (comparable to that of *M.baileyi*). The central emargination of this lobe is deep and rectangular and the entire lobe projects very slightly beyond the apices of the lateral lobes. The margins of the filament are parallel and direct, the fronds tabular, and divided by a very deep constriction into two dichotomously incised segments, the ultimate subdivisions of which are emarginate. In the Bengal variety, the teeth-like projections next to the terminal lobe are acutely angular, instead of being rounded and, as in the case of *Onychonema*, the projecting processes, by which cohesion is either secured or increased, overlap each other alternately in adjacent fronds. The cell length is 85–90 μm, width 70–75 μm, width of isthmus 11–13 μm, width of apex 32 μm, width of polar lobes 15 μm (Wallich 1860; Turner 1892).

#### Distribution.

This variety seems to be endemic to southeastern tropical Asia (Lower Bengal, India; Fig. 2 and Suppl. material 2), where it was found in the 1800’s by Wallich (Wallich 1860). A single record of this variety from Sierra Leone (Woodhead and Tweed 1958; Suppl. material 2) is doubtful, because descriptions and illustrations of the cell morphology, as well as information about the sampling site, are absent.

#### Habitat and ecology.

No information is available in the literature about the habitats or ecology of this variety.

### ﻿3. Micrasterias foliacea var. multiornata

Taxon classificationPlantaeDesmidialesDesmidiaceae

Zalocar de Domitrovic, 1981

A58C8141-8CDD-52A1-8E40-DAF71539C9CD

Micrasterias foliacea var. multiornata Zalocar de Domitrovic, 1981. “Physis (Buenos Aires)”, Sec.B, 40(98): 58, fig. 2: 13.

#### Morphology.

This variety differs from the type by the presence and distribution of warts, situated in a row from the isthmus to half the length of the sinus. There is also a wart on the basis of the upper side lobes. Cells are 120–124 μm long, 115–120 μm wide, polar lobes are 53–57 μm wide, isthmus is 20–22 μm wide (Zalocar de Domitrovic 1981).

#### Distribution.

The variety is endemic to South America and was only found in tropical areas of Argentina (Fig. 2 and Suppl. material 2 for more information).

#### Habitat and ecology.

M.foliaceavar.multiornata was found in a wetland for which no ecological data is available.

### ﻿4. Micrasterias foliacea var. nodosa

Taxon classificationPlantaeDesmidialesDesmidiaceae

(Behre) Levanets & Janse van Vuuren stat. nov.

59CE416A-83A0-523C-B1D0-DC952345A58D

M. foliacea f. nodosa Behre 1956. “Archiv für Hydrobiologie / Supplement” 23: 84, Pl. 10, fig. 5, Basionym.

#### Taxon depository.

PhycoBank registration: http://phycobank.org/103725.

Nodular cell wall thickenings are unique morphological features of this variety and therefore it is proposed to raise taxonomical status of this form to the variety.

#### Morphology.

This taxon was described by Karl Behre (1956) as “forma nodosa” based on cell morphology – it differs from the type by the presence of nodular wall thickenings. Usually, thickenings are located on the inner corners and opposed points of adjacent lobes. Sometimes adjacent lobes are connected by ridge-like thickenings. It was noted that this variety is not a teratological form, because similar wall thickenings were found in numerous cells collected in several sites. Also, no transitional forms were seen between the nodular and the typical forms and the two forms occurred side by side in some samples. Dimensions: 70–74 x 80 μm, isthmus 16 μm.

#### Distribution.

Variety *nodosa* was rarely encountered, and only recorded from a few islands in Indonesia and the Philippines (southeastern Asia; Fig. 2 and Suppl. material 2 for more information).

#### Habitat and ecology.

In all cases M.foliaceavar.nodosa were found in lake environments. In the Philippines the water bodies were characterized by clear water, covered by plants, containing detritus and other remains of higher plants. In Indonesia it was recorded from a crater lake (Suppl. material 2). No data on physico-chemical environmental variables was present for these water bodies.

### ﻿5. M. foliacea var. nurulislamii

Taxon classificationPlantaeDesmidialesDesmidiaceae

Levanets & Janse van Vuuren, 2023: 1, no fig.

15BBA397-97CA-5ECD-8EBF-8B369D3CE7D1

Micrasterias foliacea var. spinosa Islam & Ashrafi, *nom. inval*. 2004. “Bangladesh Journal of Plant Taxonomy” 6, Pl. 3, figs 12–14. Basionym.

#### Remarks.

Islam and Begum (2004) published a paper on *Micrasterias* Agardh from selected areas of Bangladesh and described a new variety of *M.foliacea* (var. spinosa). This varietal designation was invalid and was not designated in accordance with Art. 40.6 of ICN (Turland et al. 2018). In addition, the varietal name “Micrasteriasfoliaceavar.spinosa Islam & Ashrafi” was invalid as Micrasteriasfoliaceavar.spinosa Levanets and Guiry (2021) had priority. A new name, in honor of National Professor of Bangladesh, Abul Khayer Mohammed Nurul Islam, was therefore proposed for this variety (Levanets and Janse van Vuuren 2023).

#### Morphology.

This variety differs from the type because there are numerous spines on the cell wall. The cell wall is covered with many stout, curved spines, unequal in size. The incision between the lobes is usually wide open. Cell length 33–76 μm, width 34.5–77 μm, isthmus 5.0–11.5 μm (Islam and Begum 2004).

#### Distribution.

M.foliaceavar.nurulislamii was found only once in Bangladesh (Suppl. material 2).

#### Habitat and ecology.

It was found in a ditch. No ecological information is available.

### ﻿6. Micrasterias foliacea var. ornata

Taxon classificationPlantaeDesmidialesDesmidiaceae

Nordstedt, 1869

942BA409-6F62-51C4-AB8F-6FE6F56AC5EB

Micrasterias foliacea var. ornata Nordstedt, 1869. “Videnskabelige Meddelelser fra den Naturhistorisk Forening i Kjøbenhavn for Aaret” 21: 221, Taf. 2, fig. 16.

#### Morphology.

Each semicell is rectangular and the cell wall bears one to three small spines along the sinus, as well as on the upper margin of the upper lateral lobe near its base and the lower margin of the lower lateral lobes near the isthmus (Kim 2013, 2014). The presence of the spines on the upper and lower margins of the lateral lobes, as well as the incision which separates the polar lobe, is regarded as the major difference between this variety and the type (Nordstedt 1869; Zalocar de Domitrovic 1981).

In Asian specimens of both the specific form and var. ornata a peculiar phenomenon, never seen in any American specimens, was observed. This is a warping of the surface of the filament, resulting in the twisting of the side (edge) view of the chains into a sinusoidal curve which is sometimes quite pronounced. It is caused by the curving and dishing in opposite directions of the right and left lateral lobes of one semicell, those of the other semicell being curved and dished in the reverse manner (Scott and Prescott 1961). Illustrations of these twisting of the chains can be found in Scott and Prescott (1961).

#### Distribution.

Although variety *ornata* is less widespread than var. foliacea, it is also widely distributed throughout the world. Similar to the type, it was not recorded anywhere in Europe and in North America it was only recorded from northwestern Canada and two eastern states of the USA. It is more abundant in South America, Africa, and Asia and it was also recorded from Australia. The distribution of var. ornata is illustrated in Fig. 2 and more details regarding its patterns of distribution are presented in Suppl. material 3.

#### Habitat and ecology.

Variety *ornata* was recorded from a wide range of different ecological niches – it was found in different types of freshwaters, both standing (rainwater pools, rock ponds, rice fields, reservoirs, lakes, swamps, wetlands, flooded savannahs and lagoons) and flowing (creeks, moderate to rapidly flowing rivers). It was growing on the leaves of *Hymenachneamplexicaulis* (Poaceae) by Salazár Pereira (1991) and Salazar (2006/2007). It was also found in a coastal area in Nigeria (Kadiri 2002).

According to Kim (2013) this variety occurs mostly in oligo-mesotrophic, neutral-alkaline water bodies. The variety had a rare abundance in a natural oligotrophic pond on Jeju Island (South Korea), with pH ranging between 6.1 and 7.5 and conductivity between 44 and 108 μS/cm (Kim 2014). In Venezuela it was found in a flooded savannah which was acidic, with a low salinity and a high biomass of macrophytes (Salazár Pereira 1991; Salazar 2006/2007).

### ﻿7. M. foliacea var. quadrinflata

Taxon classificationPlantaeDesmidialesDesmidiaceae

Scott & Prescott, 1961

8D9EA977-5E31-5F5E-90C5-50994A065912

M. foliacea var. quadrinflata Scott & Prescott, 1961. “Hydrobiologia” 17(1–2): 48, Pl. 15, figs 5–8.

#### Morphology.

M.foliaceavar.quadrinflata differs from the type in having two large, prominent semi-ellipsoidal hollow swellings at the base of the lateral lobes, each bearing a long spine at the narrow ends. In addition, there may or may not be, four other long spines on each semicell, two adjacent to each of the swellings. Cells are 69–72 μm long and 63–72 μm wide. The isthmus is 12 μm wide and the teeth are 15–18 μm long (Scott and Prescott 1961).

#### Distribution.

The distribution of this variety is plotted in Fig. 2 (with more detailed information in Suppl. material 2). It is limited to the Hindustan and Indochina peninsulas and Indonesian archipelago and was recorded from India, Indonesia, Malaysia and Thailand.

#### Habitats and ecology.

Variety *quadrinflata* was found in freshwater lakes, rivers, reservoirs, swamps and wetlands (India, Indonesia and Malaysia), as well as in a rice paddy field in Thailand.

In Malaysia, the shallow parts and edges of the Tasek Bera swamp lake were covered with *Lepironiaarticulata* associations. Aquatic plants such as *Utricularia* spp., *Hydrilla* sp., *Nymphoidesindica* and *Pandanushelicopus* were present in still water areas, while *Utricularia* sp., *Cryptocorynegriffithii*, *Scirpusconfervoides* and *Pandanushelicopus* were present in running water (Ratnasabapathy and Kumano 1974). At the time of sampling the temperatures ranged between 23.4 and 33.4 °C (in still water) and between 23.8 and 26.9 °C (in running water) and the pH ranged between 4.7 and 5.3 Ratnasabapathy and Kumano 1974). In South Sumatra it was also found in acidic waters in Lebak Danau (pH between 5.0 and 5.5; Scott and Prescott 1961).

### ﻿8. M. foliacea var. spinosa

Taxon classificationPlantaeDesmidialesDesmidiaceae

G.A. Prowse ex Levanets & Guiry, 2021: 2.

4BB707E4-4844-57E4-AA91-0C56E6807B5B

Micrasterias foliacea var. spinosa G.A. Prowse, *nom. inval*. 1969. “Gardens’ Bulletin”, Singapore 24: 341, Pl. 4, text-fig. 2(a). Basionym.

#### Morphology.

The variety differs from all other forms because the isthmus is widely opened and due to the large width of the gap between the subterminal and terminal lobes. Pairs of prominent sharp teeth are borne on either side of the isthmus and on both sides of the base of terminal lobes. Cells 72–75 μm long., 68–70 μm wide, isthmus 8 μm wide (Prowse 1969).

#### Distribution.

Its current known distribution is limited to only one location in the Malayan peninsula (Suppl. material 2 and Fig. 2).

#### Habitat and ecology.

Variety *spinosa* was commonly found, together with var. quadrinflata, in the Tasek Bera forest swamp lake for which ecological conditions are described in the paragraph on var. quadrinflora above.

## ﻿Conclusions

Chain forming cells of *M.foliacea* were found in water samples from Botswana and Mozambique. The morphology of cells of *M.foliacea* found in these samples corresponded to that given in earlier descriptions of the type species. The species is easily distinguished from other species of the genus by its peculiar apex, interlocking the cells to form chains of up to more than 100 cells. Research on this species resulted in a review of the taxonomy, morphology, worldwide distribution, and ecology of the different varieties of *M.foliacea*, presented in this paper.

During research on the species, it was noted that doubtful records exist for different varieties of *M.foliacea*, mainly as a result of the lack of drawings (or micrographs) and new description of previously described varieties. This paper attempted to correct these mistakes and a new status is proposed, namely:

- *Micrasteriasfoliacea* var. *nodosa* (Behre) Levanets et Janse van Vuuren, stat. nov. – It is proposed to raise the taxonomical status of this form, described by Behre as *M.foliacea* Bailey ex Ralfs f. *nodosa*, to the variety, as its nodular cell wall thickenings are unique morphological features.

Detailed analysis of the distribution of *M.foliacea* and its varieties presented an interesting and clear picture, collated in a distribution map. Despite the wide geographical distribution of varieties *foliacea* (type) and *ornata*, they are completely absent from Europe and the majority of North America; both these varieties were only recorded from the eastern side of the latter continent. During this study M.foliaceavar.foliacea was observed in two new locations in southern Africa, namely Botswana and northern Mozambique. In general, most other varieties (*elongata*, *nodosa*, *spinosa*, *nurulislamii* and *quadrinflata*) are much more limited regarding their distribution and were observed and recorded mostly from southeastern tropical Asia (e.g., Indonesia, Malaysia, Philippines). Only var. multiornata is endemic to tropical South America.

A review on physico-chemisty of waterbodies in which *M.foliacea* was found, indicated that it may be present in a variety of different types of habitats in both standing and flowing water. It can tolerate a wide range of water temperature and pH. It was found in both acidic, neutral and alkaline waters all over the world. Conductivity values measured in waterbodies containing *M.foliacea* indicated that it is mostly prevalent in fresh waters and findings in estuaries and oceans may be coincidental as the result of washout during runoff. This species occurs mostly in oligo-mesotrophic conditions under conditions of relatively low turbidity.

## Supplementary Material

XML Treatment for Micrasteriasfoliacea[var.foliacea]

XML Treatment for Micrasterias foliacea var. elongata

XML Treatment for Micrasterias foliacea var. multiornata

XML Treatment for Micrasterias foliacea var. nodosa

XML Treatment for M. foliacea var. nurulislamii

XML Treatment for Micrasterias foliacea var. ornata

XML Treatment for M. foliacea var. quadrinflata

XML Treatment for M. foliacea var. spinosa

## Supplementary materials

Supplementary material 1 Geographical distribution of M.foliaceavar.foliacea throughout the world. Data type Distribution, references Explanation note Habitat types and locations are indicated, together with the reference stating its presence. https://binary.pensoft.net/file/847714This dataset is made available under the Open Database License (http://opendatacommons.org/licenses/odbl/1.0/). The Open Database License (ODbL) is a license agreement intended to allow users to freely share, modify, and use this Dataset while maintaining this same freedom for others, provided that the original source and author(s) are credited.Anatoliy Levanets, Sanet Janse van Vuuren

Supplementary material 2 Geographical distribution of M.foliaceavar.elongata, *multiornata*, *nodosa*, *nurulislamii*, *quandrinflata* and *spinosa* throughout the world Data type Distribution, references Explanation note Habitat types and locations are indicated, together with the reference stating its presence. https://binary.pensoft.net/file/847715This dataset is made available under the Open Database License (http://opendatacommons.org/licenses/odbl/1.0/). The Open Database License (ODbL) is a license agreement intended to allow users to freely share, modify, and use this Dataset while maintaining this same freedom for others, provided that the original source and author(s) are credited.Anatoliy Levanets, Sanet Janse van Vuuren

Supplementary material 3 Geographical distribution of M.foliaceavar.ornata throughout the world Data type Distribution, references Explanation note Habitat types and locations are indicated, together with the reference stating its presence. https://binary.pensoft.net/file/847716This dataset is made available under the Open Database License (http://opendatacommons.org/licenses/odbl/1.0/). The Open Database License (ODbL) is a license agreement intended to allow users to freely share, modify, and use this Dataset while maintaining this same freedom for others, provided that the original source and author(s) are credited.Anatoliy Levanets, Sanet Janse van Vuuren

Supplementary material 4 Supplementary references Data type references https://binary.pensoft.net/file/847717This dataset is made available under the Open Database License (http://opendatacommons.org/licenses/odbl/1.0/). The Open Database License (ODbL) is a license agreement intended to allow users to freely share, modify, and use this Dataset while maintaining this same freedom for others, provided that the original source and author(s) are credited.Anatoliy Levanets, Sanet Janse van Vuuren
