# Supplementary material for: ﻿Morphology, taxonomy, biogeography and ecology of Micrasteriasfoliacea Bailey ex Ralfs (Desmidiales, Zygnematophyceae)
Source: PhytoKeys. 2023 May 9;226:33–51. doi: 10.3897/phytokeys.226.103500 (PMC10189646; doi:10.3897/phytokeys.226.103500)
Supplement: Supplementary material 1 — Geographical distribution of M.foliaceavar.foliacea throughout the world. [file phytokeys-226-033_article-103500__-s001.docx]

**Supplementary References**

Alfinito S (2011) A check list of the freshwater algal flora of Sierra Leone, Tropical West Africa. I. *Cyanophyceae* to *Conjugatophyceae* (exclusive of *Bacillariophyceae*). Biodiversity Journal 2(3): 121–144.

Ali AD, Abiem I, Alisha EB, Musa PJ (2016) Floristic Composition of soft-bodied Algae of Pandam Lake (Pandam Wildlife Park, Nigeria). International Journal of Pure & Applied Bioscience 4(4): 39–49. <http://dx.doi.org/10.18782/2320-7051.2328>

Atangana Étémé R, Couté A (1985) Note sur quelques *Micrasterias* Ag. du Cameroun (*Chlorophyta*, *Zygophyceae*, *Desmidiales*). Cryptogamie: Algologie 6(2): 125–131.

Atlas of Living Australia (2022) <http://www.ala.org.au/> [accessed: 20.01.2022].

Aquino-Leite ALT (1990) *Desmidiaceae* (*Chlorophyta*) e os fatores fisico-quimicos da Lagoa Bonita, Distrito Federal, Brasil. Dissertação de mestrado, Departamento de Botânica, Universidade de Brasília: 1–102.

Bailey JW (1847) Notes on the algae of the United States. American Journal of Science and Arts, Series 2(3): 80-85, 399–403.

Barbaruah AD, Dutta DC (2014) Certain limno-chemical characteristics and commercial fish catch in Monoha beel ecosystem, Morigaon, India. The Clarion 3(2): 53–61.

Behre K (1956) Die Süβwasser-Algen der Wallacea-Expedition (ohne die Diatomeen und Peridineen). Archiv für Hydrobiologie / Supplement 23(1): 1–104.

Bernard Ch (1908) Protococcacées et Desmidiées d’eau douce, recoltées à Java. Département de l’Agriculture aux Indes Néerlandaises, Batavia: 1–230.

Bernard Ch (1909) Sur quelques algues unicellulares d’eau douce récoltées dans le domaine Malais. Département de l’Agriculture aux Indes Néerlandaises, Buitenzorg: 1–94.

Bharati SG, Pai KM (1972) Some desmids from Kodaikanal Lake, South India. Phykos 2: 27–36.

Bicudo CEM, Sormus L (1982) Desmidioflórula paulista, 2: gênero *Micrasterias* C. Agardh ex Ralfs. Bibliotheca Phycologica 57: 1–230.

Bishop JE (1973) Limnology of a small Malayan River Sungai Gombak. Dr. W. Junk B.V. Publishers, The Hague: 1–485.

Borge O (1925) Die von F.C. Hoehne Während der expedition Roosevelt-Rondon gesammelten Süsswasseralgen. Arkiv för Botanik 19(17):1–56.

Bostock PD, Holland AE (2010) Census of the Queensland Flora. Queensland Herbarium Biodiversity and Ecosystem Sciences, Department of Environment and Resource Management, Brisbane: 1–320.

Bourrelly P (1957) Algues d’eau douce du Soudan Français, region du Macina (A.O.F.). Bulletin de l’Institut fondamental d’Afrique noire, sér.A, 19(4): 1047–1102.

Bourrelly P (1964) Vll. Algae. Les algues des eaux courantes de Madagascar. Verhandlungen der Internationalen Vereinigung für Theoretische und Angewandte Limnologie 15: 758–763.

Bourrelly P (1966) Quelques algues d’eau douce de Canada. Internationale Revue der gesamten Hydrobiologie und Hydrographie 51(1): 45–126.

Bourrelly P (1975) Quelques algues d’eau douce de Guinée. Bulletin du Muséum national d’Histoire naturelle, 3^e^ série, n.276, 20: 1–72.

Bourrelly P, Couté A (1991) Desmidiées de Madagascar (*Chlorophyta, Zygophyceae*). J. Cramer in der Gebrüder Borntraeger Verlagsbuchhandlung, Berlin – Stuttgart: 1–349 (Bibliotheca Phycologica 86).

Britton NL (1889) Catalogue of plants found in New Jersey. John L. Murphy Publishing Co., Trenton, New Jersey: 1–64.

Brown HJ (1930) The Desmids of the Southeastern Coastal Plain Region of United States. Transactions of the American Microscopical Society 49(2): 97–139.

Brühl P, Biswas K (1926) Algae of the Loktak Lake. Memoirs of the Asiatic Society of Bengal 8: 257–316.

Carter N (1926) Freshwater algae from India. Records of the Botanical Survey of India 9(4): 263–302.

Chainapong T, Traichaiyaporn S (2001) Diversity of Algae Genera *Micrasterias* and *Pleurotaenium* in Muang District, Narathiwas Province. In: 27^th^ Congress on Science & Technology of Thailand, October 2001: 23004162.

Chung YH, Shim JH, Lee MJ (1965) A study on the microflora of the Han River. 1. The phytoplanktons and the effect of the marine water in the lower course of the Han River. Korean Journal of Botany 8(4): 7–29 [in Korean].

Chung YH, Kay ES, Park DW (1968) A study on the microflora of the Han River. 2. The phytoplanktons and its seasonal variation in the area of the Chun-chon and the Chung-pyong reservoir of the Han River. Korean Journal of Botany 11: 1–30.

Claassen MI (1982) ‘N taksonomiese studie van Transvaalse varswater *Euglenophyceae* en *Chlorophyceae*. PhD Thesis, University of Pretoria, South Africa: 1–542 [in Afrikaans].

Coesel PFM (2000) Desmids (*Chlorophyta, Desmidiaceae*) from Thale Noi (Thailand). Nordic Journal of Botany 20: 369–383. <https://doi.org/10.1111/j.1756-1051.2000.tb00751.x>

Colt LC (1976) Sixty-six years later, *Micrasterias* *foliacea* Bailey again in New England. Rhodora 78(816): 783–784.

Compère P (1967) Algues du Sahara et de la région du lac Tchad. Bulletin du Jardin botanique national de Belgique / Bulletin van de National Plantentuin van België 37(2): 109–288.

Compère P (1977) Algues de la région du Lac Tchad VII – Chlorophycophytes (3e partie: Desmidiées) (1). Cahiers Office de la Recherche Scientifique et Technique d'Outre-Mer, Série hydrobiologie 11: 77–177.

Couté A, Rousselin G (1975) Contribution à l’étude des algues d’eau douce du Moyen Niger (Mali). Bulletin du Muséum national d’Histoire naturelle, 3^e^ série, n.277, 21: 73–175.

Cronberg G, Gieske A, Martins E, Prince Nengu J, Stenström I-M (1995) Hydrobiological Studies of the Okavango Delta and Kwando/Linyanti/Chobe River, Botswana. I. Surface Water Quality Analysis. Botswana Notes and Records 27: 151–226.

Cushman JA (1908) A synopsis of the New England species of *Micrasterias*. Rhodora 10(114): 97–111.

Das D, Keshri JP (2013) Desmids from South Sikkim, India. Nelumbo 55: 172–180.

Das D, Keshri JP (2016) Desmids of Eastern Himalaya. J. Cramer in Borntraeger Science Publishers, Stuttgart: 1–260 (Bibliotheca Phycologica 119).

Das P, Baruah PP (2016) Seasonal variation of epiphytic algae in relation to water chemistry at Deepor beel Ramsar site. International Journal of Environmental Biology 6(1): 16–23.

Das R (2020) Preliminary checklist of desmids from Kokrajhar District, Assam, India. Journal of Biodiversity and Environmental Sciences 17(4): 10–20.

Day SA, Wickham RP, Entwisle TJ, Tyler PA (1995) Bibliographic check-list of non-marine algae in Australia. Flora of Australia, Supplementary Series 4: 1–276.

Dayala VT, Salas PM, Sujatha CH (2014) Spatial and seasonal variations of phytoplankton species and their relationship to physicochemical variables in the Cochin estuarine waters, Southwest coast of India. Indian Journal of Marine Sciences 43(6) 937–947.

Debnath M, Mandal DK (2011) Some interesting Desmids of Birbhum District, West Bengal, India. Wesleyan Journal of Research 4(1): 56–60.

Deka SJ, Sarma GS, Deka SP (2011) Preliminary Checklist of Desmids of Urpad Beel (Wetland) Goalpara District, Assam, India. Asian Journal of Experimental Biological Sciences 2(3): 391–398.

Dingley M (2003) Desmids from the genus *Micrasterias* from Australia’s tropical north. <http://www.microscopy-uk.org.uk/mag/artnov03/mddesmid.html> [accessed: 4.12.2014].

Ecology and biodiversity of Lower Ganga River basin. Farakka to Gangasagar (2012) Ganga River Basin Environment Management Plan, Indian Institutes of Technology. Report Code: 026_GBP_IIT_ENB_DAT_04_Ver_Jun 2012: 1–48.

Ekhator O, Alika F (2016) Phytoplankton diversity indices of Osse River, Edo State, Nigeria. Ife Journal of Sciences 18(1): 63–84.

Ekhator O, Ihenyen JO, Okooboh GO (2013) Desmids of Osse River, Edo State, Nigeria. International Journal of Modern Botany 3(2): 25–31. <https://doi.org/10.5923/j.ijmb.20130302.03>

Ekhator O, Opute FI, Akoma OCh (2014) A Checklist of the Phytoplankton Flora of a Southern Nigerian Lotic Ecosystem. Current Research Journal of Biological Sciences 6(1): 1–6. <http://dx.doi.org/10.19026/crjbs.6.5490>

Feitosa MF (2011) Análise comparativa da dieta, seletividade alimentar e estrutura da ictiofauna, juvenis e espécies de pequeno porte, em lagoas marginais do reservatório de Rosana (rio Paranapanema, SP/PR). Tese apresentada ao Instituto de Biociências da Universidade Estadual Paulista - UNESP, Campus de Botucatu, como parte dos requisitos para a obtenção do Título de Doutor em Ciências Biológicas, Área de concentração: Zoologia, Botucatu: 1–160.

Findlay DF, Kling HJ (1979) A species list and pictorial reference to the phytoplankton of Central and Northern Canada - Part I. Fisheries & Marine Service Manuscript Report No.1503: 1–326.

Flores CJS (2001) Taxonomy and Distribution of the Freshwater Micro-crustaceans and Green Algae of Puerto Rico, Three Contributions to American Cladocerology, and a Bibliography on West Indian Limnology. PhD Thesis (Limnology and Marine Sciences), University of Wisconsin, Madison: 1–1421.

Foerster K (1972) Desmideen aus dem Suedosten der Vereinigten Staaten von Amerika. Nova Hedwigia 23(2-3): 515–644.

Freitas LC, Loverde-Oliveira SM (2013) Checklist of green algae (*Chlorophyta*) for the state of Mato Grosso, Central Brazil. Check List 9(6): 1471–1483. <https://doi.org/10.15560/9.6.1471>

Frietas JF, Kamat ND (1979) Desmidiaceae of Nagpur. Phykos 18(1-2): 97–103.

Fritsch FE (1907) A general consideration of the subaërial and fresh-water algal flora of Ceylon. A contribution to the study of tropical algal ecology. Part I. - subaërial algae and algae of the inland fresh-waters. Proceeding of the Royal Society, London, Series B, 79: 197–254.

Frohne W (1939) *Anopheline* breeding: Suggested classification of ponds based on characteristic desmids. U.S. Public Health Reports 54(30): 1363–1387.

Fujisawa K (1936) On Japanese *Micrasterias* Ag. 1827 found in Japan. Natural History Magazine (Hakubutu) 34(58): 12–17.

Furtado JI, Nori S (1982) Tasek Bera. The Ecology of a Freshwater Swamp. Dr. W. Junk BV Publishers, The Hague - Boston – London: 1–413 (Monographiae Biologicae 47).

Gama LRM, Sousa MM, Almeida ICS, Caridade EO, Ferreira-Correia MM, Terceiro AM (2011) Microfitoplâncton das baías do Golfão Maranhense e Litoral Oriental do Estado do Maranhão. Boletim do laboratório de hidrobiologia 24(1): 13–26.

Gerrath JF, Denny P (1989) Freshwater Algae of Sierra Leone VI. Desmids (*Gonatozygon* to *Pleurotaenium*) from the Southern Province. Nova Hedwigia 48: 167–186.

Gomes PP (2007) Variação espacial e temporal da comunidade fitoplanctônica da Lagoa Bonita, DF. Dissertação apresentada ao Departamento de Ecologia do Instituto de Ciências Biológicas da Universidade de Brasília, como requisito parcial à obtenção do grau de Mestre em Ecologia: 1–66.

Gontcharov AA (1997) Contribution to the desmid flora of the Primorsky Territory, Russia. Bulletin of National Science Museum, Ser. B., Tokyo 23(2): 59−80.

González AC (2009) Catálogo de las algas y cianoprocariotas dulciacuícolas de Cuba. Impreso en Universidad de Cienfuegos, Cuba: 1–147.

Grönblad R (1945) De Algis brasiliensibus, praecipue Desmidiaceis, in regione inferiore fluminis Amazonas a Prof. August Ginzberger (Wien) anno MCMXXVII collectis. Acta Societatis Scientiarum Fennicae, Serie B, Helsingforsiae 2(6): 1–43.

Gutwiński R (1902) De algis a Dre M. Raciborski anno 1899 in insula Java collectis. Bulletin international de l'Académie des sciences de Cracovie 9: 575–617.

Hà LTT, Dũng NV (2010) Microalgae in Boc Nguyen reservoirs, Ha Tinh province. Tạp chí khoa học, tập 39, số 4A: 20–27 [in Vietnamese].

Hahina AG (1948) Microbial flora of Bolon’ Lake in the relation to the nutrition of the Silver carp. Proceedings of the Pacific Research Institute of Fishery and Oceanography 27: 187–219 [in Russian].

Halder N (2016) Impact of nutrients on distribution of *Micrasterias* sp. in Hooghly Province, West Bengal, India. International Journal of Environment 5(2): 36–47. <https://doi.org/10.3126/ije.v5i2.15004>

Hall JD, Fučíková K, Chien LO, Lewis LA (2010) An assessment of proposed DNA barcodes in freshwater green algae. Cryptogamie, Algologie 31(4): 529–555.

Hasan MA (2012) *Chlorophyceae* and their changes in relation to some abiotic factors in Chalan-beel waters, Bangladesh. Bangladesh Journal of Progressive Science & Technology 10(2):141–146.

Higashi M (1938) *Micrasterias* from Japan. Japanese Journal of Limnology (Rikusuigaku Zasshi) 8(3-4): 428–433 [in Japanese].

Hirano M (1959) Flora Desmidiarum Japonicarum V. Contributions from the Biological Laboratory, Kyoto University 7: 226–301.

Hirano M (1960) Flora Desmidiarum Japonicarum VII. Contributions from the Biological Laboratory, Kyoto University 11: 387–474.

Hirano M (1967) Freshwater algae collected by the Joint Thai-Japanese Biological Expedition to Southeast Asia 1961-62. In: Kira T, Iwata W (Eds) Nature and Life in Southeast Asia. Vol.V. Published by Fauna and Flora Research Society, Kyoto: 1–71.

Hirano M (1972) Desmids from Cambodia, with special reference to Phytoplankton of Lake Grands Lacs (Tonle Sap). Contributions from the Biological Laboratory, Kyoto University 22(3-4): 123–157.

Hirano M (1975) Phytoplankton from Lake Boraphet in the central Plain of Thailand. Contributions from the Biological Laboratory, Kyoto University 24(4): 187–203.

Hu HJ, Li YY, Wei YX, Zhu HZ, Chen JY, Shi ZX (1980) The Freshwater Algae of China. Shanghai Science and Technology Press, Shanghai: 1–525 [in Chinese].

Hu H, Wei Y (2006) The freshwater algae of China. Systematics, taxonomy and ecology. Science Press, Beijing: 1–1023 [in Chinese].

Iltis A (1980) Les algues. In: Durand JR, Levêque C (Eds) Flore et faune aquatiques del’Afrique sahélo-soudanienne 1. Office de la Recherche Scientifique et Technique d'Outre-Mer, Paris: 9–61.

Imazu T (1979) Phytoplankton in small irrigation ponds of Kanzaki District, Hyogo Prefecture. Japanese Journal of Limnology 40(2): 93–101 [in Japanese].

iNaturalist contributors, iNaturalist 2023 iNaturalist Research-grade Observations. iNaturalist.org. Occurrence dataset https://doi.org/10.15468/ab3s5x accessed via GBIF.org on 2023-04-14. https://www.gbif.org/occurrence/3902520602

Irénéé-Marie F (1938‘1939’) Flore desmidiale de la région de Montréal. Frères de l’Instruction chrétienne, Laprairie: 1–547.

Irénéé-Marie F (1942) Étude de la flore desmidiale de la région du lac St-Jean. Le Naturaliste canadien 69: 248–259.

Irénéé-Marie F (1951) Desmidiées de la région de Québec. Le Naturaliste canadien 78(7-8): 177–221.

Irénéé-Marie F (1952) Contribution à la connaissance des Desmidiées de la région du Luc-St-Jeans. Hydrobiologia 4(1-2): 1–208.

Irénéé-Marie F (1957) Les micrasterias de la région des Trois-Rivières. Hydrobiologia 9(1): 66–88.

Irénéé-Marie F (1959) Expedition Algologique dans la nord de la Mauricie, basin de Mattawin. Hydrobiologia 13(4): 319–381.

Islam AKMN (1970) Contributions to the knowledge of desmids of East Pakistan, Part I. Nova Hedwigia 20(3-4): 903–983.

Islam AKMN, Begum A (2004) Desmids from some selected areas of Bangladesh: 1. Genus *Micrasterias* Agardh. Bangladesh Journal of Plant Taxonomy 11(2): 1–14.

Islam AKMN, Ifranullah HM (2006) Hydrobiological studies within the tea gardens at Srimangal, Bangladesh. V. Desmids (*Euastrum, Micrasterias*, *Actinotaenium* and *Cosmarium*). Bangladesh Journal of Plant Taxonomy 13(1): 1–20. <https://doi.org/10.3329/bjpt.v13i1.589>

Jacobs J (1968) A preliminary check list of the freshwater algae in South Carolina. Journal of the Elisha Mitchell Scientific Society 84: 454–457.

Jena M, Adhikary SP (2011) Algal diversity of Loktak Lake, Manipur. Nelumbo 53: 21–48.

John J, Francis MS (2013) An Illustrated Algal Flora of Kerala. Vol. 1. Idukki District. Pranatha Books, Cohin: 1–345.

Johnson LN (1894a) On Some Species of *Micrasterias*. Botanical Gazette 19(2): 56–60.

Johnson LN (1894b) Some New and Rare Desmids of the United States - I. Bulletin of the Torrey Botanical Club 21(7): 285–291.

Joshua W (1886) Burmese Desmidieae, with descriptions of new species occurring in the neighbourhood of Rangoon. Journal of the Linnean Society of London, Botany 21: 634–655.

Kadiri MO (2002) A checklist of desmids in Nigeria. Global Journal of Pure and Applied Sciences 8(2): 223–237. <https://doi.org/10.4314/gjpas.v8i2.16036>

Kadiri MO, Opute FI (1989) A rich flora of *Micrasterias* from the Ikpoba Reservoir, Nigeria. Archiv für Hydrobiologie 116(3): 391–399.

Kalita SR, Ahmed R, Das M (2016) Physico-Chemical Characteristics and Phytoplanktonic Diversity of Urpod Beel, Goalpara Assam (India). Series a Thesis Conceptual Magazine 3(7): 10–14 [journal title in Hindi: श्रृंखला एक शोधपरक वैचारिक पत्रिका - Shrinkhla Ek Shodhparak Vaicharik Patrika].

Kamat ND (1975) Algae of Vidarbh, Maharashtra. Journal of the Bombay Natural History Society 72(2): 450–476.

Kanetsuna Y (1962) Limnological studies of Mizoroga-ike Pond in Kyoto, with especial reference to the plankton and algae (Diatomaceae and Desmidiaceae). Japanese Journal of Limnology (Rikusuigaku Zasshi) 23(3-4): 113–132 [in Japanese].

Kasrina SI, Jayanti WE (2012) Diversity of microalgae in the swamp water on Bengkulu City, Bentiring Permai District, as an alternative source of learning biology in senior high school. Jurnal Exacta 10(1): 36–44 [in Indonesian: Ragam jenis mikroalga di air rawa kelurahan Bentiring Permai kota Bengkulu sebagai alternatif sumber belajar biologi SMA].

Karthick B, Ramachandra TV (2006) Water quality status of Sharavathi River basin, Western Ghats. Sahyadri Conservation Series - 5, Envis Technical Report 23: 1–63.

Kerkar V, Lobo A (2009) Desmid diversity for Northern Goa, India. In: Proceedings of the International Conference on Algal Biomass, Resources and Utilization: 132–141.

Kim HS (2013) Algal Flora of Korea. Volume 6, Number 3. *Charophyta: Conjugatophyceae* (Desmids II). *Desmidiales: Closteriaceae* II, *Desmidiaceae* II. Freshwater Green Algae*.* Sang Pal Lee Publisher: 1–103.

Kim HS (2014) Desmids from Korea; 1. *Desmidiaceae* 1 (*Micrasterias*). Journal of Ecology and Environment 37(4): 285–298. <https://doi.org/10.5141/ecoenv.2014.032>

Kim HS (2018) Diversity of phytoplankton species in Cheonjin Lake, northeastern South Korea. Journal of Ecology and Environment 42(29): 1–19. <https://doi.org/10.1186/s41610-018-0080-5>

Konan ES, Da KP, Aka NM (2012) Etude systématique des: *Conjugatophyceae*, *Chlorophyceae* et *Euglenophyceae* d’une lagune tropicale: la lagune de Fresco (Côte d’Ivoire). Journal of Applied Biosciences 49: 3406–3414.

Kossinskaya EK (1960) *Desmidiales*. 1. Flora of Spore Plants of USSR. Vol.5. Conjugatae (2). Moscow, Leningrad, Academy of Sciences of USSR Publishing House: 1–706 [in Russian: Коссинская, Е.К. 1960. Флора споровых растений СССР. Том 5. Коньюгаты или сцеплянки (2). Москва, Ленинград: Издательство Академии Наук СССР: 706 c.].

Kumar BN, Choudhary SK (2009) Algal flora of Jagatpur wetland in the middle Ganga flood plain near Bhagalpur, Bihar (India). Journal of the Indian Botanical Society 88: 8–11.

Lackey J, Lackey E (1967) A partial checklist of Florida freshwater algae and protozoa with reference to McCloud and Cue Lakes. Bulletin Series 131, Florida Engineering and Industrial Experiment Station, College of Engineering, University of Florida, Gainesville: 1–28.

Lalèyè P, Chikou A, Gnohossou P, Vandewalle P, Philippart JC, Teugels G (2006) Studies on the biology of two species of catfish *Synodontis schall* and *Synodontis nigrita* (*Ostariophysi*: *Mochokidae*) from the Ouémé River, Bénin. Belgian Journal of Zoology 136(2): 193–201.

Lamkom T, Kaewlurn V (2001) Diversity of Plankton in the Pak Mun Reservoir. In: Proceedings of the 39th Conference of Kasetsart University on Fish Science: 70–79 [in Thai: ธนาทิพย์ แหลมคม และ วิชาญ แก้วเลื่อน. 2543. การศึกษาความหลากหลายของแพลงก์ตอนพืชและแพลงก์ตอนสัตว์ในพื้นที่ อ่างเก็บนํ้าของเขื่อนปากมูล. *การประชุมทางวิชาการของมหาวิทยาลัยเกษตรศาสตร์ ครั้งที่ 39 สาขาประมง*: 70–79].

Lee AL (2013) Diversity of phytoplankton in Lake Chini, Pahang, Northern Chenahan. <http://www.ukm.my/ahmad/tesispelajar/fitochenahan.htm> [accessed: 24.04.2015] [in Malay: Kepelbagaian fitoplankton di Laut Chenahan, Tasik Chini, Pahang].

Leentvaar P (1975) Hydrobiological observations in Surinam with special reference to the man-made Brokopondo Lake. Studies of the fauna of Suriname and other Gayanas 56: 1–173.

Lemmermann E (1905) Über die von Herrn Dr. Walter Volz auf seiner Weltreise gesammelten Süsswasseralgen. Abhandlungen herausgegeben vom Naturwissenschaftlichen Verein zu Bremen 18: 143–174.

Lenzenweger R (1974) Beitrag zur Desmidiaceen-Flora von West-Borneo. Mitteilungen der botanischen Arbeitsgemeinschaft am Oberösterreichischen Landesmuseum Linz 6/2: 103–128.

Lewis WM (1978) A compositional, phytogeographical and elementary structural analysis of the phytoplankton in a tropical lake: Lake Lanao, Philippines. Journal of Ecology 66: 213–226.

Li WKW, Andersen RA, Gifford DJ, Incze LS, Martin JL, Pilskaln CH, Rooney-Varga JN, Sieracki ME, Wilson WH, Wolff NH (2011) Planktonic Microbes in the Gulf of Maine Area. PLOS ONE 6(6), e20981: 1–13. <https://doi.org/10.1371/journal.pone.0020981>

Lin ZW, Lin S, Gu JG, Hu C (2013) Responses of phytoplankton community to the construction of small hydropower stations in Hainan Province. Acta Ecologica Sinica (Shengtai Xuebao) 33(4): 1186–1194 [in Chinese]. <https://doi.org/10.5846/stxb201205300790>

Ling HU, Taylor PA (2000) Australian Freshwater Algae (exclusive of diatoms). J. Cramer in der Gebrüder Borntraeger Verlagsbuchhandlung, Berlin, Stuttgart: 1–643. (Bibliotheca Phycologica, Band 105).

Lorch DW, Engels M (1979) Observations on filament formation in *Micrasterias* *foliacea* (*Desmidiaceae*, *Chlorophyta*). Journal of Phycology 15: 322–325.

Lütkemüller J (1900) Desmidiaceen aus den Ningpo-Mountains in Centralchina. Annalen des K.K. Naturhistorischen Hofmuseums 15(2): 115–126.

Maheswari K, Baluswami M (2017) Taxa of *Desmidaceae* from Tamil Nadu, India – *Micrasterias* Agardh. Indian Hydrobiology 17(1): 57–70.

Mama AC, Mbeng OL, Dongmo ChT, Moto I, Ngoupayou NJR, Ohondja ALM (2016) Tidal variations and its impacts on the abundance and diversity of phytoplankton in the Nyong Estuary of Cameroon. Journal of Multidisciplinary Engineering Science and Technology 3(1): 3667–3675.

Marazzi L (2014) Biodiversity and Biomass of Algae in the Okavango Delta (Botswana), a Subtropical Flood-Pulsed Wetland. PhD Thesis, Department of Geography, University College London: 1–420.

Marçal SF (2005) Variação espacial e sazonal da abundância relativa do fitoplâncton na baía do Coqueiro (Pantanal de Poconé, Mato Grosso, Brasil). Unpublished Undergraduate dissertation, Universidade Federal de Mato Grosso, Cuiabá: 1–53.

Marie-Jean Eudes S (1969) La flore de Rawdon. Contributions de l’Institut botanique de l’Université de Montréal 79: 41–71.

Martin MR (2013) Cedar Eden Environmental, LLC. <http://www.cedareden.com/micro/chloro.html> [accessed: 20.04.2015].

Martins-Da-Silva RCV, Bicudo CEM (2007) Lista das algas planctônicas (exclusive diatomáceas) do lago Água Preta, município de Belém, Estado do Pará. In: Gomes JI, Martins MB, Martins-Da-Silva RCV, Almeida SS (Eds) Mocambo: diversidade e dinâmica biológica da área de pesquisa ecológica do Guamá (Apeg). Belém, PA, Museu Paraense Emílio Goeldi, Embrapa Amazônia Oriental: 1–454.

Martínez Almeida V (1989) Contribuciones a la revisión del género *Micrasterias* (Desmidiaceae: Chlorophyta) en Cuba. Acta Botanica Cubana 77: 1–14.

McAlice BJ (1975) Preliminary checklist of planktonic microalgae from the Gulf of Maine. Maine Sea Grant Information Leaflet 9, March 1975 (MEU-H-75-001), Cooperative Extension Service, University of Maine, Orono, Maine: 1–17.

Medvedeva LA (2007) Results of algological study of middle part of River Bureya basin. In: Medvedeva LA, Teslenko VA, Tiunova TM (Eds) Hydro-ecological monitoring in Bureyskaya Hydro-Electric Power Station zone influences. Institute of Water and Ecological Problems of Far East Branch of Russian Academy of Sciences, Khabarovsk: 64–79 [in Russian].

Medvedeva LA, Nikulina TV (2014) Catalogue of freshwater algae of the southern part of the Russian Far East. Vladivostok, Dal’nauka Publishing House: 1–271 [in Russian: Каталог пресноводных водорослей юга Дальнего Востока России. Владивосток, Дальнаука: 271 с.].

Medvedeva LA, Nikulina TV (2019) Species diversity of cyanobacteria and algae in the Bureya River basin (Khabarovsk Territory). In: Vladimir Ya. Levanidov’s Biennial Memorial Meetings. Vol. 8. Vladivostok: Dalnauka Publishing House: 91–113 [in Russian: Видовое разнообразие цианобактерий и водорослей водоемов бассейна р. Бурея (Хабаровский край) / Чтения памяти Владимира Яковлевича Леванидова. Вып. 8. – Владивосток: Дальнаука, 2019: 91–113].

Miranda J, Krishnakumar G (2015) Microalgal diversity in relation to the physicochemical parameters of some Industrial sites in Mangalore, South India. Environmental Monitoring and Assessment 187(11), article 664: 1–25. <https://doi.org/10.1007/s10661-015-4871-1>

Mukherjee D, Srivastava DN (1993) Some desmids of Purulia (West Bengal). Journal of the Indian Botanical Society 72: 293–297.

Mungkung R, Pengthamkeerati P, Chaichana R, Watcharothai S, Kitpakornsanti K, Tapananont S (2014) Environmental impacts of organic rice farming in Thailand by using life cycle assessment to support policy decision on sustainable agriculture. In: EcoBalance 2014. The 11^th^ International Conference on EcoBalance. 27-30 October 2014, Tsukuba, Japan. Abstract Book: 28C4–2.

Ngearnpat N (2009) Biodiversity of desmids in some freshwater resources of Thailand and its correlation with water quality. PhD Thesis, The Graduate School, Chiang Mai University: 1–255.

Ngearnpat N, Coesel PFM, Peerapornpisal Y (2008) Diversity of desmids in three Thai peat swamps. Biologia 63/6: 897–902. <https://doi.org/10.2478/s11756-008-0140-x>

Ngearnpat N, Peerapornpisal Y (2007) Application of desmid diversity in assessing the water quality of 12 freshwater resources in Thailand. Journal of Applied Phycology 19(6): 667–674. <https://doi.org/10.1007/s10811-007-9191-6>

Ngo H, Prescott GW, Czarnecki DB (1986-1987) Additions and confirmations to the algal flora of Itasca State Park. I. Desmids and diatoms from North Deming Pond. Journal of the Minnesota Academy of Science 52(2): 14–26.

NIES-Collection. List of Strains. Seventh Edition. Microalgae and Protozoa (2004) Research Report from the National Institute for Environmental Studies, Japan, No. 182, R-182-2004: 1–256.

Nikulina TV (2016) The algal flora of streams from the Tsaplichya Lagoon Basin of Amur Bay (Primorye, Khasan District). In: Freshwater Life. Vol. 2. Vladivostok: Dalnauka Publishing House: 70–87 [in Russian: Альгофлора водотоков бассейна лагуны Цапличья Амурского залива (Приморский край, Хасанский район). Жизнь пресных вод. Вып. 2. Владивосток: Дальнаука: 70–87].

Nishikawa Y, Mizuno T (1969) On the *Micrasterias* of ponds in the Southern Part of Osaka prefecture. The Bulletin of the Japanese Society of Phycology (Sôrui) 17(1): 4–10 [in Japanese].

Noor S, Salleh A, Nasrodin S (2012) Desmid flora in Tasik Sungai Semuji, Kuantan, Pahang, Malaysia. In: Proceedings of the 2^nd^ International Conference on Arts, Social Sciences & Technology, Penang, Malaysia, 3^rd^-5^th^ March 2012, Paper Code No. I2040: I2040-1 – I2040-10.

Nordstedt CFO (1869) Desmidiaceae. In: Warming E (Ed) Symbolae ad floram Brasiliae centralis cognoscendam. Videnskabelige Meddelelser fra den Naturhistorisk Forening i Kjøbenhavn for Aaret 1869 14, 15: 195–234.

Nordstedt CFO (1880) De algis et Characeis. 1. De algis nonnullis praecipue Desmidieis, inter Utricularias Muséi Lugdono-Batavi. Acta Universitatis Lundensis 16: 1–14.

Okada Y (1936a) Notes on Japanese desmids, with special reference to the newly found species. I. The Botanical Magazine 50(590): 79–85.

Okada Y (1936b) Notes on Japanese Desmids, with special reference to the newly found species. III. The Botanical Magazine 50(594): 313–317.

Okada Y (1943) On the Desmids of Okinawa Island. Acta phytotaxonomica et geobotanica 13: 261–273 [in Japanese].

Okada Y (1952) Taxonomical studies on the genus *Micrasterias* with special reference to the species of Japan and its surrounding areas. Memoirs of the Faculty of Fisheries, Kagoshima University 2(1): 93–140 [in Japanese].

Okogwu OI, Ugwumba AO (2012) Response of phytoplankton functional groups to fluctuating water level in two shallow floodplain lakes in Cross River, Nigeria. Inland Waters 2: 37–46. <https://doi.org/10.5268/IW-2.1.466>

Okogwu OI, Ugwumba AO (2013) Seasonal dynamics of phytoplankton in two tropical rivers of varying size and human impact in Southeast Nigeria. International Journal of Tropical Biology and Conservation/Revista de Biología Tropical 61(4): 1827–1840.

Oliveira IB de, Moura CWN, Bicudo CEM (2009) *Micrasterias* C. Agardh ex Ralfs (*Zygnematophyceae*) de duas Áreas de Proteção Ambiental da planície litorânea do norte da Bahia, Brasil. Revista Brasileira de Botanica 32(2): 213–232. <https://doi.org/10.1590/S0100-84042009000200003>

Opute FI (1992) Contribution to the knowledge of algae of Nigeria. 1. Desmids from the Warri / Forcados Estuaries. Part II. The genera *Euastrum* and *Micrasterias*. Algological Studies 65: 73–92.

Opute FI, Kadiri MO (2013) *Phytoplankton algae of Nigeria. A practical & theoretical guide. Volume 1: The desmids.* Mindex Publishing Company Limited, Benin City: 1–304.

Oyadomari J (2013) Keweenaw Algae. <http://www.keweenawalgae.mtu.edu/gallery_pages/charophyceans3.htm> [accessed: 20.04.2015].

Parameswaran S, Radhakrishnan S, Selvaraj C, Bhuyan BR (1971) Fish yield from Assam pond kept under different experimental conditions. Indian Journal of Fisheries 18(1&2): 67–83.

Paudel N (2017) New record of desmids from Ramwell-Rhino Lake, Chitwan, Nepal. International Journal of Science and Research 6(8): 523–526. <https://doi.org/10.21275/ART20175881>

Peerapornpisal Y, Suphan S, Ngearnpat N, Pekkoh J (2008) Distribution of chlorophytic phytoplankton in Northern Thailand. Biologia 63(6): 852–858. <https://doi.org/10.2478/s11756-008-0112-1>

Pham MN, Tan HTW, Mitrovic S, Yeo HHT (2011a) A checklist of the algae of Singapore. Raffles Museum of Biodiversity Research, National University of Singapore, Singapore: 1–100.

Pham MN, Tan HTW, Mitrovic S, Yeo HHT (2011b) A checklist of the algae of Singapore, 2^nd^ Edition. Raffles Museum of Biodiversity Research, National University of Singapore, Singapore: 1–99.

Pham TL, Phan DD (2011) Initial record of community structure of phytoplankton in Lang Sen wetland reserve, Long An Province. In: Proceedings of the 4^th^ National Conference on Ecology and Biological Resources, Hanoi, Vietman, 21 October 2011, Agriculture Publishing: 198-205 [in Vietnamese: Ghi nhận ban đầu về khu hệ thực vật nổi ở khu bảo tồn đất ngập nước Láng Sen, Tỉnh Long An. Hội nghị khoa học toàn quốc về sinh thái và tài nguyên sinh vật lần thứ 4: 198–205].

Phan VM (2010) Diverse groups of aquatic organisms in Trang An Resorts, Ninh Binh Province. In: Research Center of Natural Resources and Environment. Proceedings of the Second National Conference for Environment and Sustainable Development, Science and Technology Publisher: 131–146 [in Vietnamese: Đa dạng các nhóm thủy sinh vật, khu du lịch Tràng An Ninh Bình. Trung Tâm Nghiên cứu Tài nguyên và Môi trường, kỷ yếu hội thảo quốc gia lần thứ II Môi trường và phát triển bền vững, 2010. Nxb. Khoa học Kỹ thuật: 131–146].

Phillips JA (2002) Algae. In: Henderson RJF (Ed) Names and distribution of Queensland plants, algae and lichens. Queensland Government Environmental Protection Agency, Brisbane: 228–244.

Pillai TG, Sollows LD (1980) Cage Culture of Fish in Nepal. A report prepared for the Integrated Fisheries and Fish Culture Development Project, Nepal, Rome, FAO, FI: DP/NEP/73/025, Field Document 8: 1–28.

Ponce HG, Hernandez SG, Aviles MG, Galan RG, Talavera ML (1992) Informe de hidrobiologia Rio San Juan. Informe of Centro para la investigacion en recursos acuaticos de Nicaragua. Junio 20, Managua-Nicaragua: 1–13.

Prakash J, Kishore S, Asthana DK, Misra PK, Singh SK (2005) Morphotaxonomy of fresh water chlorophycean algae (desmids) from Siddharth Nagar, U.P., India. Ecoprint 12: 21–25. <https://doi.org/10.3126/eco.v12i0.3193>

Prasad BN, Mehrotra RK (1977) Some desmids new to Indian flora. Journal of the Indian Botanical Society 56: 343–350.

Prasad BN, Misra PK (1985) Genus *Micrasterias* Agardh from Andamans. Geophytology 15(1): 33–38.

Prasad BN, Misra PK (1992) Freshwater algal flora of Andaman and Nicobar Islands, vol. II. Singh B. and Singh M.P. Publication, Dehradun, India: 1–284.

Prasertsin T, Peerapornpisal Y (2018) Distribution and isolation of microalgae for lipid production in selected freshwater reservoirs of northern Thailand. Biodiversitas 19(1): 343–350. <https://doi.org/10.13057/biodiv/d190147>

Prescott GW, Croasdale HT, Vinyard WC (1977) A synopsis of North American Desmids. Part 2, sect.2. University of Nebraska Press, Lincoln: 1–413.

Prescott GW, Scott AM (1943) The desmid genus *Micrasterias* Agardh in southeastern United States. Papers of the Michigan Academy of Science, Arts & Letters 28: 67–82.

Prescott GW, Scott AM (1952) The Algal Flora of Southeastern United States. V. Additions to Our Knowledge of the Desmid Genus *Micrasterias* 2. Transactions of the American Microscopical Society 71(3): 229–252.

Prowse GA (1962) Distributional relationships of Malayan freshwater algae. In: Proceedings of the Ninth Pacific Science Congress of the Pacific Science Association 1957, Volume 4, Botany. Published by the Secretariat, Ninth Pacific Science Congress, Department of Science, Bangkok, Thailand: 217–218.

Prowse GA (1969) Some new desmid taxa from Malaya and Singapore. Gardens’ Bulletin, Singapore 24: 337–346.

Rai SK, Misra PK (2008) On some desmids from Koshi Tappu wildlife reserve, Nepal. Ecoprint 15: 47–58. <https://doi.org/10.3126/eco.v15i0.1942>

Rai SK, Rai RK, Paudel N (2008) Desmids from Bees-hazaar Lake, Chitwan, Nepal. Our Nature 6: 58–66. <https://doi.org/10.3126/on.v6i1.1656>

Ratnasabapathy M, Kumano S (1974) Desmids from Tasek Bera, West Malaysia. The Bulletin of the Japanese Society of Phycology (Sôrui) 22(1): 22–28 [in Japanese].

Ribeiro CA, Moura CWN (2012) Levantamento da biodiversidade de *Micrasterias* (Zygnematophyceae) do Pantanal Dos Marimbus (Baiano e Remanso), Chapada Diamantina, Bahia, Brasil. In: 63º Congresso Nacional de Botânica acontecerá no período de 11 a 16 de novembro de 2012 no Centreventos Cau Hansen em Joinville, SC. Seção. Sistemática/Taxonomia: 34.

Ribeiro CA, Ramos GJP, Oliveira IB, Moura CWN (2015) *Micrasterias* (Zygnematophyceae) de duas áreas do Pantanal dos Marimbus (Baiano e Remanso), Chapada Diamantina, Bahia, Brasil. Ciências Biológicas, Sitientibus série 15: 1–12. <https://doi.org/10.13102/scb578>

Richardson N (1973) Freshwater algae from the Dade-Collier Jetport. Florida Scientist 36(2-4): 205–208.

Riehl W, Infante A, Masa Y (1987) Desmidias del embalse de Guri, Venezuela. Acta Científica Venezolana 38: 106–121.

Rino JA (1971) Contribuição para o conhecimento das algas de água doce de Moçambique - II. Revista de Ciências Biológicas, Série A, 4: 9–55.

Sagala EP (2019) The study of diversity index on plankton community in Lematang River to determinate the quality of waters as habitat of local fishes. IOP Conference Series: Earth and Environmental Science 374(012019): 1–9. <https://doi.org/10.1088/1755-1315/374/1/012019>

Salazar C (2007‘2006’) Desmidiaceae (Zygophyceae) asociadas a *Hymenachne amplexicaulis* (Poaceae) en una sabana tropical inundable, Venezuela. Memoria de la Fundación La Salle de Ciencias Naturales 166: 95–131.

Salazár Pereira CV (1991) Estudio sistemático de la ficoflora perifítica (Desmidiales y Euglenaceae) asociada a Himenachne amplexicaulis en una sabana inundable de Venezuela. Doctorado en Ciencias Naturales, Universidad Nacional de La Plata, Facultad de Ciencias Naturales y Museo, La Plata: 1–237.

Salisbury RK (1936) The desmids of Florida. Ohio Journal of Science 36(1): 55–61.

Sanchez AL (1993) Étude du phytoplankton des lacs d'Inondation du bas Orénoque. Relations avec les facteurs du milieu. Thèse de Doctorat de l’Université Paul Sabatier de Toulouse, Spécialité*:* Écologie des Systemes Aquatiques Continentaux, Toulouse, 1992/10/30. Office de la Recherche Scientifique et Technique d'Outre-Mer, Paris: 1–227.

Sanilkumar MG, Thomas KJ (2006) Diversity and seasonal variation of algae in Muriyad wetland (Part of Vembanad-Kol wetlands – a Ramsar site). Journal of Economic and Taxonomic Botany 30(3): 656–666.

Santos MA, Bicudo CEM, Moura CW (2018) Taxonomic notes on the species of the genus *Micrasterias* (Desmidiaceae, Conjugatophyceae) from the Metropolitan Region of Salvador, Bahia, Brazil. Checklist 14(6): 1027–1045. <https://doi.org/10.15560/14.6.1027>

Santos MA dos, Bicudo CEM, Moura CWN (2016) O gênero *Micrasterias* (*Desmidiaceae*, *Conjugatophyceae*) na Ilha de Itaparica, Bahia. Brazil. Iheringia, Série Botânica, Porto Alegre 71(3): 296–315.

Sarim FM (1980) A monograph on the desmids of Pakistan. PhD Thesis, Department of Botany, University of Peshawar, Pakistan: 1–286.

Schmidle W (1902) Berichte über die botanischen Ergebnisse der Nyassa-See und Kinga-Gebirgs-Expedition der Hermann- und Elise- geb. Heckmann-Wentzel-Stiftung. V. Algen, insbesondere solche des Plankton, aus dem Nyassa-See und seiner Umgebung, gesammelt von Dr. Fülleborn. Botanische Jahrbücher 32: 56–88.

Schumacher G (1956) A qualitative and quantitative study of the plankton algae in southwestern Georgia ponds. American Midland Naturalist 56: 88–115.

Scott AM, Prescott GW (1961) Indonesian Desmids. Hydrobiologia 17(1-2): 1–132.

Shao K-T, Peng Ch-I, Wu W-J (2010) Taiwan Species Checklist. Forestry Bureau, Council of Agriculture, Executive Yuan, Taiwan: 1–932.

Sharma BK, Sharma S (2021) Phytoplankton diversity of a subtropical reservoir of Meghalaya state of northeast India. Aquatic Sciences and Engineering 36(2), 51–65. <https://doi.org/10.26650/ASE2020740218>

Sheeba S (1999) Certain aspects of the ecology of Ithikkara River. PhD Thesis, Mahatma Ghandi University, Zoology Research Centre, C.M.S. College, Kottayam: 1–408.

Sheeba S (2009) Biotic environment and sand mining - a case study from Ithikkara river, South West coast of India. Journal of Industrial Pollution Control 25(2): 133–138.

Silva FKL da, Felisberto SA (2015) *Euastrum* and *Micrasterias* (family Desmidiaceae) in lentic tropical ecosystem, Brazil. Biota Neotropica 15(1): 1–12. <https://doi.org/10.1590/1676-06032015007914>

Silveira AMJr (2012) Composição e biomassa microfitoplanctônica associadas a variáveis fisico e químicas em dois transectos da zona estuarinado Rio Amazonas (Amapá, Amazônia, Brasil). Dissertação apresentada ao Programa de Pós-Graduação em Ciências da Saúde da Universidade Federal do Amapá - UNIFAP, como parte dos requisitos necessários para a obtenção do título de Mestre em Ciências da Saúde; Área de Concentração, Ensaios Biológicos, Macapá: 1–92.

Skinner S, Townsend SA (2005) Macro-algae in the streams of the Darwin Region, and the Daly River catchment. Report 12/2005D. Northern Territory Government, Department of Infrastructure, Planning and Environment: 1–44.

Smith GM (1924) Phytoplankton of the Inland Lakes of Wisconsin. Part II. Desmidiaceae. Bulletin of the University of Wisconsin, Serial No. 1270, General Series No. 1048: 1–227.

Stone GE (1900) Flora of Lake Quinsigamond. Annual Report of Worcester Natural History Society 1900: 3–8.

Swift DR (1984) Periphyton and water quality relationships in the Everglades Water Conservation Areas. In: Gleason PJ (Ed) Environments of South Florida. Present and Past. II. Miami Geological Society, Coral Gables, Florida: 97–117.

Tahirou DI (2013) Les algues du fleuve Niger et des milieux humides connexes de l’ouest du Niger. Thèse unique de doctorat de l’Université Abdou Moumouni: 1–202.

Tan M-Ch, Zhong Z-X, Bao Sh-K, Wang M-Sh (1984) The desmids in Dai Lake of Jinyun Mountain, Beibei, Chongqing (A continued report). Studies on the Chongqing algae flora II. Journal of the Southwest Teachers College 6(2): 82–93 [in Chinese].

Tarar JL, Charjan U, Bodhke S (1998) Contribution the knowledge of desmids from Nagpur. Phykos 37: 59–67.

Taylor WR (1935) The fresh-water Algae of Newfoundland. Pt. II. Papers of the Michigan Academy of Science, Arts & Letters 20: 185–230.

Therezien Y (1985) Contribution a l’Etude des Algues d’Eau Douce de la Bolivie. Nova Hedwigia 41(1-4): 505–576.

Thomas JK, Sreekumar S, Jaya Cheriyan (2003) Muriyad Wetlands: ecological changes and human consequences. Project report submitted to Kerala Research Programme on Local Development, Centre for Developmental Studies, Thiruvanandapuram: 1–83.

Thomasson K (1960) Notes on the plankton of Lake Bangweulu. Part 2. Nova Acta Regiae Societatis Scientiarum Upsaliensis, Ser.IV, 17(12): 1–43.

Thomasson K (1965) Notes on algal vegetation of Lake Kariba. Nova Acta Regiae Societatis Scientiarum Upsaliensis, Ser.IV, 19(1): 1–34.

## Thomasson K (1966) Le phytoplancton du lac Shiwa Ngandu: Phytoplankton of Lake Shiwa Ngandu. Exploration hydrobiologique du bassin du lac Bangweolo et du Luapula, v.4, fasc.2: 1–91.

Thomasson K (1971) Amazonian Algae. Mémoires de l’Intitut Royal des Sciences naturelles de Belgique, Ser 2, Bruxelles, n.86: 1–57.

Thomasson K (1977) Two conspicuous desmids from Amazonas. Botaniska Notiser, Stockholm 130: 41–51.

## Thomasson K (1986) Algal Vegetation in North Australian Billabongs. Nova Hedwigia 42(204): 301–378.

Tiodolf AM, Stålnacke P (2009) A limnological study of the Sesan River in Cambodia in the dry season: focus on toxic cyanobacteria and coliform bacteria. STRIVER Technical Brief, Issue N.12: 1–11.

Tran Ngoc Duc (2002) Study of the composition and distribution of phytoplankton in the Vam Co Tay River in Long An Province. Thesis of the Master of Science, ecology code: 01:05:20. The course instructor Nguyen Thanh Tung III. Ho Chi Minh City: 1–67 [in Vietnamese: Trần Ngọc Đức 2002. Nghiên cứu thành phần và phân bố phiêu sinh thức vật trên sông Vàm Cỏ Tây thuộc tỉnh Long An*. Luận án thạc sỹ khoa học chuyên ngành: sinh thái học mã số: 01.05.20. Người hướng dẫn khoa hoc ts. III Nguyễn Thanh Tùng, thành phố Hồ Chí Minh*].

Tropical Lake Ecology Assessment with Emphasis on Changes in Salinity of Lakes (2004) Project No. SAA-140714. Technical Memorandum # 2. Inventory of Flora and Fauna. Prepared by: URS Holdings, Inc. for Panama Canal Authority: 1–82.

Turner WB (1892) Algae Aquae Dulcis Indiae Orientalis. The Fresh-Water Algae (principally Desmidieae) of East India. Kongl. Boktryckeriet, Stockholm: 1–187.

Ubong G, Aniema I-E, Ndueso I (2017) Bio-monitoring and diversity of phytoplankton in a tropical estuarine mangrove swamp in Akwa Ibom State, South-South, Nigeria. European Journal of Biotechnology and Bioscience 5(4): 71–79.

Vaas KF, Sachlan M (1948) On the Ecology of some small Lakes near Buitenzorg, Java. Hydrobiologia 1(1-4): 238–250.

van Oye P (1922) Contribution à la connaissance de la flore et de la faune microscopiques des Indes Néerlandaises. Annales de biologie lacustre 11(1): 130–151.

van Oye P (1947) Desmidiaceeën der omgeving van Matadi in verband met hun verspreiding in Belgisch Congo. Biologische Jaarbücher 14: 145–157.

van Oye P (1953) Contribution à la connaissance des Desmidiées du Congo belge. Hydrobiologia 5(3): 239–308.

van Vuuren L (2014) Research builds body of knowledge to protect Waterberg rivers. The Water Wheel March/April 2014: 8–11.

Varela R, Varela M, Fariña AC (1983) Microalgas del Bajo Orinoco y Delta Amacuro, Venezuela. I. Cyanophyceae, Euglenophyceae, Xantophyceae, Euchlorophyceae, Zygophyceae. Memoria Sociedad de Ciencias Naturales La Salle 120(43): 59–88.

Vijayan D, Ray JG (2015) Green algae of a unique tropical wetland, Kuttanadu, Kerala, India, in relation to soil regions, seasons, and paddy growth stages. International Journal of Science, Environment and Technology 4(3): 770–803.

Vyverman W (1991) Desmids from Papua New Guinea. J. Cramer in der Gebrüder Borntraeger Verlagsbuchhandlung, Berlin – Stuttgart: 1–201 (Bibliotheca Phycologica 87).

Vyverman W (1992) Altitudinal Distribution of Non-cosmopolitan Desmids and Diatoms in Papua New Guinea. British Phycological Journal 27: 49–63.

Wallich GC (1860) Description of Desmidieae from Lower Bengal. Annals and Magazine of Natural History, Series 3, 5: 184-197, 273–285.

Wei Y (1996) Desmids from southern bays of the Donghu Lake, Wuhan, China. Acta Phytotaxonomica Sinica 34(6): 653–671 [in Chinese].

Wei YX (2003) Flora algarum sinicarum aquae dulcis, Tomus VII. *Chlorophyta*, *Zygnematales*, *Mesotaeniaceae*, *Demidiales*, *Desmidiaceae*, Section I. Science Press, Beijing: 1-267.

West W, West GS (1898) On some desmids of the United States. The Journal of the Linnean Society, Botany 33(231): 279–322.

West W, West GS (1902) A Contribution to the Freshwater Algae of Ceylon. The Transactions of the Linnean Society of London, Second Series - Botany 6(3): 123–215.

West W, West GS (1907) Fresh-water algae from Burma, including a few from Bengal and Madras. Annals of the Royal Botanic Garden, Calcutta 6: 175–260.

Whelden RM (1942) Notes on New England algae. II. Some interesting New Hampshire algae. Rhodora 44(521): 175–187.

Whitford L (1943) The fresh-water algae of North Carolina. Journal of the Elisha Mitchell Scientific Society 59(2): 131–170.

Wildeman DE (1900) Les Algues de la Flore de Buitenzorg (Essai d’une Algologique de Java). Librairie et imprimerie ci-devant E.J. Brill, Leide: 1–457.

Williamson DB (1994) A contribution to knowledge of the desmid flora of South Africa and the adjoining states of Ciskei and Swaziland*. Archiv für Hydrobiologie / Supplement* 99(4): 415–487.

Williamson DB (1998) Desmids from Peninsula Malaysia. Algological Studies 90: 45–77. <https://doi.org/10.1127/algol_stud/90/1998/45>

Williamson DB, Marazzi L (2013) A new *Cosmarium* (*Chlorophyta*, *Desmidiaceae*) variety from the Okavango Delta, Botswana. Quekett Journal of Microscopy 42: 35–37.

WRM (2007) Floodplain Impact Study: Assessment of Ecological Health of Control and Exposed Forested Oxbow Lakes Using Plankton and Aquatic Invertebrate Assemblages. Unpublished report by Wetland Research & Management to Ok Tedi Mining Limited, July 2007: 1–80.

Wolle F (1882) Fresh-water algae. VI. Bulletin of the Torrey Botanical Club 9(3): 25–30.

Wolle F (1884) Desmids of the United States and list of American Pediastrums with eleven hundred illustrations on fifty-three colored plates. Moravian Publication Office, Bethlehem, Pennsylvania: 1–182.

Wolle F (1892) Desmids of the United States and list of American Pediastrums with nearly fourteen hundred illustrations on sixty-four colored plates. Moravian Publication Office, Bethlehem, Pennsylvania: 1–182.

Wołoszyńska J (1913) O glonach planktonowych niektórych jezior jawańskich, z uwzględnieniem glonów Sawy. - Das Phytoplankton einiger javanischer Seen mit Berücksichtigung des Sawa-Planktons. Bulletin international de l'Académie des sciences de Cracovie: 649–709.

Woodhead N, Tweed RD (1958) A check-list of tropical West African algae. Hydrobiologia 11(3-4): 299–395.

Woodhead N, Tweed RD (1960) A second check-list of tropical West African algae. Hydrobiologia 15(3): 225–286.

Woodson B, Holoman V (1964) A systematic and ecological study of algae in Chesterfield County, Virginia. Virginia Journal of Science 15: 51–70.

Yossan S, Moonsin P (2015) Using dominant phytoplankton as a bioindicator of water quality in Huay Samran, Sisaket Province. Research & Development Bank Ltd. Publications 38(3): 295–309 [in Thai: สิริพร ยศแสน & และ ปริญญา มูลสิน (2558): การใช้แพลงก์ตอนพืชชนิดเด่นในการบ่งชี้คุณภาพน้ำในห้วยสำราญ จังหวัดศรีสะเกษ. วารสารวิจัยและพัฒนา มจธ. ปีที่ 38 ฉบับที่ 3 กรกฎาคม - กันยายน 2558: 295–309].

Zalocar de Domitrovic Y (1981) Desmidiales (*Chlorophyta*) de la provincia de Corrientes (Argentina). II. El género *Micrasterias*. Physis (Buenos Aires), Sec. B, 40(98): 55–62.
