# Supplementary material for: ﻿Morphology, taxonomy, biogeography and ecology of Micrasteriasfoliacea Bailey ex Ralfs (Desmidiales, Zygnematophyceae)
Source: PhytoKeys. 2023 May 9;226:33–51. doi: 10.3897/phytokeys.226.103500 (PMC10189646; doi:10.3897/phytokeys.226.103500)
Supplement: Supplementary material 2 — Geographical distribution of M.foliaceavar.elongata, multiornata, nodosa, nurulislamii, quandrinflata and spinosa throughout the world [file phytokeys-226-033_article-103500__-s002.docx]

**Supplementary Table 1**. Geographical distribution of *M. foliacea* var. *foliacea* throughout the world. Habitat types and locations are indicated, together with the reference stating its presence.

| **CONTINENT /COUNTRY/ STATE** | **HABITAT TYPE/LOCATION** | **REFERENCES** |
| --- | --- | --- |
| **AFRICA:** |  |  |
| Benin | In the stomachs of catfish, Ouémé River | Lalèyè et al. 2006 |
| Botswana | Lechwe Pan, Okavango Delta, Boro Region | Williamson and Marazzi 2013; Marazzi 2014 |
|  | Okavango Delta | Cronberg et al. 1995 |
|  | Okavango Delta, near Shakawe | Levanets and Janse van Vuuren, new record |
| Cameroon | El Béid River | Compère 1967 |
|  | Estuary of Nyong River, Cameroon coastal zone | Mama et al. 2016 |
| Chad | Lake Chad | Compère 1977 |
| Côte d’Ivoire | Fresco Lagoon | Konan et al. 2012 |
| Dem. Republic of Congo | Pond, Road from Matadi to Seke Banza | Van Oye 1947 |
|  | Swamp, Ndjili Municipality, Tshangu District | Van Oye 1953 |
| Guinea | Irrigation Canal, Koubi near Pita | Bourrelly 1975 |
| Madagascar | Between *Sphagnum*, Moramanga | Bourrelly and Couté 1991 |
|  | Lake Debo, floodplain of Niger River, Macina (Masina) region | Bourrelly 1957 |
|  | Bourem, Middle Niger River | Couté and Rousselin 1975 |
|  | Flooded meadow, Bamba | Couté and Rousselin 1975 |
|  | Alamba-Guindé | Couté and Rousselin 1975 |
|  | No details | Woodhead and Tweed 1960 |
|  | Mamery River, near Ebakika (Fort-Dauphin area) | Bourrelly 1964 |
| Mozambique | Small pond, Namaacha, Maputo Province | Rino 1971 |
|  | Palma, Cabo Delgado, south of the Tanzanian border | Levanets and Janse van Vuuren, new record |
| Niger | Niger River | Tahirou 2013 |
| Nigeria | Ikpoba Reservoir, Benin, Bendel State | Kadiri and Opute 1989; Kadiri 2002 |
|  | Warri/Forcados Estuaries, southern Nigeria | Opute 1992; Kadiri 2002; Opute and Kadiri 2013 |
|  | Coastal area | Kadiri 2002 |
|  | Ossa River, Edo State | Ekhator et al. 2013, 2014; Ekhator and Alika 2016 |
|  | Ehoma and Iyieke Lakes, Cross River | Okogwu and Ugwumba 2012 |
|  | Asu and Cross Rivers | Okogwu and Ugwumba 2013 |
|  | Pandam Lake, Plateau State | Ali et al. 2016 |
|  | Tropical estuarine mangrove swamp, Qua Iboe River estuary | Ubong et al. 2017 |
| Sierra Leone | No details | Woodhead and Tweed 1958 |
|  | Rivers: Jong and Sewa, Lakes: Gambia, Popei and Tibi | Gerrath and Denny 1989; Alfinito 2011 |
| South Africa | Marshes along Nyl River, Naboomspruit | Claassen 1982 |
|  | Mokolo River, Limpopo Province | van Vuuren 2014 |
| Tanzania | Ikapu Crater Lake | Schmidle 1902 |
| Zambia | Lakes Bangweulu, Wumba, Chaya | Thomasson 1960, 1965 |
|  | Lake Shiwa Ngandu (also known as Young Lake) | Thomasson 1966 |
|  | Lake near Songwe, Songwe River, Chimene River | Thomasson 1965 |
| Zimbabwe | Masumo River | Thomasson 1965 |
| **ASIA:** |  |  |
| Bangladesh | Dhaka (former Dacca), Rajshahi, Comilla | Islam 1970 |
|  | Maulvibazar Lake, Srimangal | Islam and Ifranullah 2006 |
|  | Chalan-beel Wetland | Hasan 2012 |
| Cambodia | Kg Prahoe River | Hirano 1972 |
|  | Sesan River | Tiodolf and Stålnacke 2009 |
| China | On *Utricularia flexuosa*, Ningpo Mountains | Lütkemüller 1900 |
|  | Freshwater | Hu et al. 1980; Wei 2003; Hu and Wei 2006 |
|  | Dai Lake, Jinyun Mountain, Beibei, Chongqing | Tan et al. 1984 |
|  | Changhua River, Hainan Province | Lin et al. 2013 |
| India | On *Utricularia* sp., Khasia | Turner 1892 |
|  | Loktak Lake, Manipur State | Brühl and Biswas 1926 |
|  | Kundil River, Assam Valley near Sadiya | Carter 1926 |
|  | Joysagar ponds, Sigsagar, Assam | Parameswaran et al. 1971 |
|  | Kodaikanal Lake, Tamil Nadu | Bharati and Pai 1972 |
|  | Ramsagar Lake, Bhandara District; Tiroda, Maharashtra State | Kamat 1975 |
|  | Uttar Pradesh State | Prasad and Mehrotra 1977 |
|  | Nagpur, Maharashtra State | Frietas and Kamat 1979; Tarar et al. 1998 |
|  | Andaman and Nicobar Islands | Prasad and Misra 1985, 1992 |
|  | Purulia District, West Bengal | Mukherjee and Srivastava 1993 |
|  | Ithikkara River, Kerala State | Sheeba 1999, 2009 |
|  | Muriyad Kayal Wetlands, Kerala State | Thomas et al. 2003; Sanilkumar and Thomas 2006 |
|  | Tal Kunda Pond, Siddharth Nagar District, Uttar Pradesh State | Prakash et al. 2005 |
|  | Freshwater, Northern Goa | Kerkar and Lobo 2009 |
|  | Jagatpur Wetland, Bihar | Kumar and Choudhary 2009 |
|  | Pond, Birbhum District, West Bengal | Debnath and Mandal 2011 |
|  | Urpad Beel Wetland, Agia, Goalpara District, Assam State | Deka et al. 2011; Kalita et al. 2016 |
|  | Epiphytic on aquatic weed, Manipur State | Jena and Adhikary 2011 |
|  | Bitang-cho and Mamencho Lakes, Delo reservoir, Kalimpong, Namchi Region, Sikkim Province | Das and Keshri 2013, 2016 |
|  | Lower Ganga River basin (marine zone) | Ecology and biodiversity of Lower Ganga River basin 2012 |
|  | Cochin Estuary, southwest coast | Dayala et al. 2014 |
|  | Monoha beel near Morigaon, Assam Province | Barbaruah and Dutta 2014 |
|  | Upper Kuttanadu wetland, Kerala State (in soils of paddy fields) | Vijayan and Ray 2015 |
|  | Deepor beel, environs of Guwahati, Assam | Das and Baruah 2016 |
|  | Pond, Hooghly, West Bengal | Halder 2016 |
|  | Periyavoorani Lake, Amoor, Madurai District, Tamil Nadu State;Veera Magaliamman Temple Pond, Keelavaniyangudi, Sivagangai District, Tamil Nadu State | Maheswari and Baluswami 2017 |
|  | Kokrajhar District, Assam State | Das 2020 |
|  | Nongmahir Reservoir, Ri-Bhoi District, near Shillong City, Meghalaya State | Sharma and Sharma 2021 |
| Indonesia | Bogor (formerly Buitenzorg) botanical garden, Java | Nordstedt 1880; Bernard 1908, 1909 |
|  | Ditches and swamps, Java | Wildeman 1900 |
|  | Lake Tjibenong near Bogor, west Java | Gutwiński 1902 |
|  | Demangan Pond, central Java | Wołoszyńska 1913 |
|  | Lake Ratoedjaja, Tjitajam (at Bogor, formerly Buitenzorg), Java | Wołoszyńska 1913 |
|  | Lake Gedeh, Tasikmalaya, west Java | Van Oye 1922 |
|  | Mangga Bolong, Bogor (formerly Buitenzorg), Java | Vaas and Sachlan 1948 |
|  | Lake Sawangan, Bogor (formerly Buitenzorg), Java | Vaas and Sachlan 1948 |
|  | Kapita River, Sanghir/Sangihe Archipelago | Behre 1956 |
|  | Timampoe Swamp near Lake Towuti, Sulawesi | Behre 1956 |
|  | Lake, eastern Borneo | Scott and Prescott 1961 |
|  | Swamp, Bentiring Permai District, Bengkulu City, southwest Sumatra | Kasrina and Jayanti 2012 |
|  | Lematang River, Lahat Region, South Sumatra | Sagala 2019 |
| Japan | Murayama-Chosuichi Reservoir, Tokyo | Fujisawa 1936 |
|  | Lake Ogura, Kyôto, Yamashito Province | Okada 1936a, 1952; Higashi 1938 |
|  | Ditch near Musashiranzan, Musashi Province | Okada 1936b, 1952; Higashi 1938 |
|  | Okinawa Island, Okinawa Prefecture | Okada 1943, 1952 |
|  | Mountain paddy field, Kei Kanai | Okada 1952 |
|  | Tomakomai, Iburi, Hokkaido | Hirano 1959 |
|  | Chûkawa-ike, Uzen, Hondo | Hirano 1959 |
|  | Ishigaki-ike, Ise, Hondo | Hirano 1959 |
|  | Shinohara-ike & Fuse-ike, Oomi | Hirano 1959 |
|  | Mizoroga-ike, Yamashiro | Hirano 1959 |
|  | Ahira, Ôsumi, Kiúshiu | Hirano 1959 |
|  | Hokkaido, Tohoku, Kanto, Kink | Hirano 1960 |
|  | Mizoroga-ike Pond, Kyoto | Kanetsuna 1962 |
|  | Ponds, southern part of Osaka | Nishikawa and Mizuno 1969 |
|  | Small irrigation pond, Himeji City, Kanzaki District, Hyogo Prefecture | Imazu 1979 |
|  | Higashihiroshima, Hiroshima | NIES-Collection 2004; Hall et al. 2010 |
| Malaysia | Sungai Gombak River | Bishop 1973 |
|  | Tasek Bera Wetland | Ratnasabapathy and Kumano 1974; Furtado and Nori 1982 |
|  | Near Melaka (Melacca) City | NIES-Collection 2004 |
|  | Tasik Sungai Semuji Lake, Kuantan, Pahang State | Noor et al. 2012 |
|  | Lake Chini, Pahang, northern Chenahan | Lee 2013 |
| Myanmar | Pond, Rangoon, estuary of Irrawa | Joshua 1886 |
|  | Epiphytic on *Utricularia fasciculata*, Burmah | Turner 1892 |
|  | Marsh, Mansang (near Hsipaw) | West and West 1907 |
| Nepal | Begnas and Rupa Lakes | Pillai and Sollows 1980 |
|  | Bees-hazaar Lake, Tikauli-3, Gitanagar VDC, Chitwan | Rai et al. 2008 |
|  | Titrigachi Pond, Koshi Tappu Wildlife Reserve, Sapta Koshi River, Sunsari and Saptari Districts | Rai and Misra 2008 |
|  | Ramwell-Rhino Lake, Barandabhar Corridor Forest, Chitwan National Park | Paudel 2017 |
| Papua New Guinea | Lakes and swamps | Vyverman 1991, 1992 |
|  | Kuambit Oxbow, Bosset Lagoon, Lake Daviumbu, Fly River Basin, western Province | WRM 2007 |
| Pakistan | Naguman River, Peshawar District; near Bannu, Khyber Pakhtunkhwa Province | Sarim 1980 |
| Phillipines | Lake Laguna de Bay, Luzon | Behre 1956 |
|  | Lake Balut | Behre 1956 |
|  | Lake Mainit | Behre 1956 |
|  | Lake Butig around Lanao Lake | Behre 1956 |
|  | Lanao Lake, Mindanao Island, southern Phillippines | Lewis 1978 |
| Russia | Bolon Lake, Amur River basin, Primorsky Territory | Hahina 1948; Kossinskaya 1960 |
|  | Small swampy lakes and ponds near Riazanovka Station, Primorsky Territory | Gontcharov 1997 |
|  | Unnamed lake, River Urgal, Khabarovsk Region | Medvedeva 2007; Medvedeva and Nikulina 2019 |
|  | Freshwater, Khabarovsk and Primorsky Territory | Medvedeva and Nikulina 2014 |
|  | Stream, Tsaplichya lagoon, Amur Bay, Khasan District, Primorsky Territory | Nikulina 2016 |
| Singapore | Ponds, botanical garden of Singapore | Lemmermann 1905; Pham et al. 2011a, b |
| South Korea | Han River, Hanchu, Noryangjin and Chuncheon, Seoul | Chung et al. 1965, 1968 |
|  | Dongbaekdongsan Wetland and oligotrophic rock pond, Jeju Island | Kim 2013, 2014 |
|  | Cheonjin Lake, Gangwon Province | Kim 2018 |
| Sri Lanka | Kosgoda and Urahaighasmahendai Paddyfields, Heneratgodha | West and West 1902 |
|  | Pools and ditches, Uplands | Fritsch 1907 |
| Taiwan | No details | Shao et al. 2010 |
| Thailand | Pond in botanical garden, Chieng Mai City, northwestern Thailand | Hirano 1967 |
|  | Irrigation Ditch, Trang City, southern Thailand | Hirano 1967 |
|  | Lake Boraphet (Bung Boraphet), confluence of Mae Nam Pin and Mae Nam Nan Rivers, Nakhon Sawan Province | Hirano 1975 |
|  | Thale Noi Wetland, Phattalung Province | Coesel 2000 |
|  | Freshwater, Muang District Narathiwas Province | Chainapong and Traichaiyaporn 2001 |
|  | Lotus Pond in Wannakadee botanical garden Chiang-Mai Province | Ngearnpat and Peerapornpisal 2007 |
|  | Laempagarung peat swamp, Phung-Nga Province | Ngearnpat et al. 2008 |
|  | Huay Luang Dam, Lampang Province | Peerapornpisal et al. 2008 |
|  | Ditch, Sukhothai Province | Peerapornpisal et al. 2008 |
|  | Nong Keaw Reservoir, Chiang Rai Province | Ngearnpat 2009 |
|  | Mae Jok Luang Reservoir and pond in Wannakadee botanical garden, Chiang Mai Province | Ngearnpat 2009 |
|  | Kwan Payao Reservoir, Phayao Province | Ngearnpat 2009 |
|  | Nong Han Kumpawapee Lake, Udon Thani Province | Ngearnpat 2009 |
|  | Mai Kaow peat swamp, Phuket Province | Ngearnpat 2009 |
|  | Tung Khai botanical garden, Trang Province | Ngearnpat 2009 |
|  | Leam Pakarung peat swamp, Phang-Nga Province | Ngearnpat 2009 |
|  | Naresuan Dam, Phitsanulok Province | Ngearnpat 2009 |
|  | Huay Samran, Sisaket Province | Yossan and Moonsin 2015 |
|  | Nong Bua Reservoir, Chang Rai, Rajabhat University, Mueang District, Chiang Rai Province | Prasertsin and Peerepornpisal 2018 |
| Vietnam | Vam Co Tay River, Long An Province | Tran Ngoc Duc 2002 |
|  | Boc Nguyen Reservoirs, Ha Tinh Province, | Hà and Dũng 2010 |
|  | Pond, Trang An Resort, Ninh Binh Province | Phan 2010 |
|  | Lang Sen Wetland Reserve, Long An Province | Pham and Phan 2011 |
| **AUSTRALIA:** |  |  |
| Northern Territory | Open water & littoral zone, Billabongs (Nankin, Jabiluka, Mine Valley, Ja Ja, Island, Winnamurra, Buffalo, Leichhardt); Magela Creek catchment; Darwin subcoastal plain; Alligator River region | Thomasson 1986; Dingley 2003 |
|  | Mitchell Creek, Darwin area | Skinner and Townsend 2005 |
|  | Katherine River | Skinner and Townsend 2005 |
| New South Wales | No details | Day et al. 1995; Ling and Taylor 2000 |
| Queensland | Cook District | Phillips 2002, Bostock and Holland 2010 |
|  | Creek near Heathlands Ranger Station, Cook Local Government Area | Atlas of Living Australia 2013 |
| **CENTRAL AMERICA:** |  |  |
| Cuba | Lakes: El Jovero and Los Indios Chicos, Pinar del Río Province;  Dams: La Fe, Isla de la Juventud; Dam Cristal, Atanagildo and Dam Vietnam near Finca El Abra.  Swamps: Ciénaga de Lanier | Martínez-Almeida 1989; González 2009 |
|  | Lake Santa María, San Luis, Pinar del Río Province | González 2009 |
|  | Oxidation pond near Lake Blanquizal, Pinar del Río Province | González 2009 |
|  | Fish Pond, Finca El Abra, Isla de la Juventud | González 2009 |
|  | Dam near Finca El Abra, Isla de la Juventud | González 2009 |
|  | Ponds, Dam Cristal, Atanagildo, Isla de la Juventud | González 2009 |
|  | Pond between Colony Hotel and Coastguard Post, Isla de la Juventud | González 2009 |
|  | El Abra Dam, Isla de la Juventud | González 2009 |
|  | Wet soil, San Felipe Plateau, Camaguey Province | González 2009 |
| Nicaragua | River San Juan | Ponce et al. 1992 |
| Panama | Gatun Lake | Tropical Lake... 2004 |
| Puerto Rico | Freshwater | Flores 2001 |
| **NORTH AMERICA:** |  |  |
| **Canada:** |  |  |
| Newfoundland | Pools, Lookout Mountain | Taylor 1935; Prescott et al. 1977 |
| Nova Scotia | No details | Prescott et al. 1977 |
| Ontario | Dawson Pond | Prescott et al. 1977 |
| Quebec | Montréal | Irénéé-Marie 1938‘1939’, 1957 |
|  | Lake St-Jean | Irénéé-Marie 1942, 1951, 1952, 1957 |
|  | Trois-Rivières, Mauricie Region | Irénéé-Marie 1957 |
|  | Unnamed Lake, basin of Mattawin River, Mauricie Region | Irénéé-Marie 1959 |
|  | Lac des Femmes, Parc du Mont Tremblant | Bourrelly 1966 |
|  | Rawdon | Marie-Jean Eudes 1969 |
|  | Gulf of Maine | McAlice 1975; Li et al. 2011 |
| **USA:** |  |  |
| Alabama | Auburn | Brown 1930; Prescott et al. 1977 |
| Conneticut | Shore of Long Pond | iNaturalist contributors, iNaturalist 2023 |
| Florida | Riverview and Childs | Salisbury 1936 |
|  | McCloud and Melrose Lakes | Lackey and Lackey 1967; Foerster 1972 |
|  | Everglades water conservation areas | Swift 1984 |
| Georgia | Ponds: (Big Cypress, Cane Water, Porter, Putney, Rays and Springfield) | Schumacher 1956 |
|  | Rushing Pond, Bulloh County | Frohne 1939 |
|  | South Magnolia Spring, Jenkins County | Frohne 1939 |
| Louisiana | Hickory Town | Prescott and Scott 1943, Prescott et al. 1977 |
| Massachusetts | Lake Quinsigamond, Worcester County | Stone 1900 |
|  | Gilder Pond, Mt. Everett, Mount Washington, Berkshire County | Wolle 1882, 1884, 1892 |
|  | Icehouse Pond, Hopkinton, Middlesex County | Colt 1976 |
| Michigan | Twelve Mile Lake, Keweenaw Peninsula | Oyadomari 2013 |
| Minnesota | North Deming Pond, Itasca State Park | Ngo et al. 1986-1987 |
| New Hampshire | Lake Winnepesaukee, Laconia | West and West 1898 |
|  | Pudding Pond, Laconia, Carroll County | Cushman 1908 |
|  | Downing Pond, New Durham | Whelden 1942 |
|  | Rocky Pond, Hollis | Martin 2013 |
| New Jersey | Denmark Pond, Morris | Britton 1889 |
| New York | Belmont Pond, Babylon | Brown 1930 |
|  | Lake Kanawauke, Orange County | Hall et al. 2010 |
|  | Large ponds, at Cold Spring Harbour, Long Island | Johnson 1894a, b |
| North Carolina | Pond, upper coastal plain, Wake County | Whitford 1943 |
| Rhode Island | Worden’s Pond near Providence | Bailey 1847 |
| South Carolina | Lotus Pond, Savannah River, Jasper County | Frohne 1939 |
|  | No details | Jacobs 1968 |
| Texas | Small private lake, west of Highway 59, south of Nacogdoches | Lorch and Engels 1979 |
| Virginia | No details | Woodson and Holoman 1964 |
| Wisconsin | Des Moines | Smith 1924 |
| **SOUTH AMERICA:** |  |  |
| Argentina | Wetlands of Iberá, Lake Trin, Province Corrientes | Zalocar de Domitrovic 1981 |
| Bolivia | River Ibaré, near Trinidad, Amazonian Bolivia | Therezien 1985 |
| Brazil | Maranhense gulf bays and Eastern coastal region, Maranhão State | Gama et al. 2011 |
|  | Tapajós River, Lake Jurucuí, Pará State | Thomasson 1971 |
|  | Flooded meadow, Terra Santa, Nhamundá region, Pará State | Thomasson 1977 |
|  | Aparecida-Pindamonhangaba, São Paulo State | Bicudo and Sormus 1982 |
|  | Pond, Guarantiguetá, São Paulo State | Bicudo and Sormus 1982 |
|  | Pond next to Highway BR-116, São Paulo State | Bicudo and Sormus 1982 |
|  | Village Conde do Pinhal, São Carlos, São Paulo State | Bicudo and Sormus 1982 |
|  | Lagoa Bonita, Estação Ecologíca de Águas Emendadas, Federal District | Aquino-Leite 1990, Gomes 2007 |
|  | Mato Grosso State | Marçal 2005; Freitas and Loverde-Oliveira 2013 |
|  | Lake Água Preta, Belém, Pará State | Martins-Da-Silva and Bicudo 2007 |
|  | Environment Protection Area Rio Capivara and Lagoas de Guarajuba, Camaçari, Bahia State | Oliveira et al. 2009 |
|  | Lagoons, Rosana Reservoir, Paranapanema River, São Paulo State/State of Paraná | Feitosa 2011 |
|  | Baiano and Remanso, Pantanal do Marimbus, Chapada Diamantina, Bahia State | Ribeiro and Moura 2012 |
|  | North and south Channel of the Amazon River estuarine ecosystem, Amapá State | Silveira 2012 |
|  | Marimbus do Baiano, Andaraí, Chapada Diamantina, Bahia State | Ribeiro et al. 2015 |
|  | Samambaia Reservoir, Federal University of Goiás, Goiánia, Goiás State | Silva and Felisberto 2015 |
|  | Municipalities of Camaçari, Mata de São João, and Vera Cruz, Bahia State | Santos et al. 2016, 2018 |
| Suriname | Brokopondo Lake, near Beerdotti; Suriname River | Leentvaar 1975 |
| Venezuela | Orinoco Delta, Bolívar State | Varela et al. 1983 |
|  | Guri Dam, Bolívar State | Riehl et al. 1987 |
|  | Orinoco and Caroni Rivers | Sanchez 1993 |
|  | Flooded savannah, Apure State | Salazár Pereira 1991; Salazár 2006‘2007’ |
