# Supplementary material for: ﻿Morphology, taxonomy, biogeography and ecology of Micrasteriasfoliacea Bailey ex Ralfs (Desmidiales, Zygnematophyceae)
Source: PhytoKeys. 2023 May 9;226:33–51. doi: 10.3897/phytokeys.226.103500 (PMC10189646; doi:10.3897/phytokeys.226.103500)
Supplement: Supplementary material 3 — Geographical distribution of M.foliaceavar.ornata throughout the world [file phytokeys-226-033_article-103500__-s003.docx]

**Supplementary Table 2**. Geographical distribution of *M. foliacea* var. *elongata*, *multiornata,* *nodosa, nurulislamii*, *quandrinflata* and *spinosa* throughout the world. Habitat types and locations are indicated, together with the reference stating its presence.

| **CONTINENT/ COUNTRY/ STATE** | **HABITAT TYPE/LOCATION** | **REFERENCES** |
| --- | --- | --- |
| ***M. foliacea* var. *elongata*** | | |
| **AFRICA:** |  |  |
| Sierra Leone | No details | Woodhead and Tweed 1958 |
| **ASIA:** |  |  |
| India | Elephant Tank, Raneegunge, Lower Bengal | Wallich 1860 |
| ***M. foliacea* var. *multiornata*** | | |
| **SOUTH AMERICA:** |  |  |
| Argentina | Wetlands Iberá, Lake Fernández, Corrientes Province | Zalocar de Domitrovic 1981 |
| ***M. foliacea* var. *nodosa*** | | |
| **ASIA:** |  |  |
| Indonesia | Crater Lake, Sasarong Lake, Sulawesi Island | Behre 1956 |
| Phillipines | Danao Lake, Leyte Island | Behre 1956 |
|  | Dagiangan Lake, Mindanao Island | Behre 1956 |
| ***M. foliacea* var. *nurulislamii*** | | |
| **ASIA:** |  |  |
| Bangladesh | Ditch near Kaliganj Village, Gazipur District | Nurul Islam and Begum 2004 |
| ***M. foliacea* var. *quadrinflata*** | | |
| **ASIA:** |  |  |
| India | Sharavathi River basin, Western Ghats | Karthick and Ramachandra 2006 |
|  | Idukki District, Kerala State | John and Francis 2013 |
|  | Gurupur River, Baikampady Industrial Area, Dakshina Kannada District, Karnataka State | Miranda and Krishnakumar 2015 |
| Indonesia | Tembaga Swamp near Djakarta City | Scott and Prescott 1961 |
|  | Palembang, Lebak Danau and Teloko Danau, South Sumatra | Scott and Prescott 1961 |
| Malaysia | Tasek Bera Wetland and forest swamp lake | Ratnasabapathy and Kumano 1974 |
| Thailand | Plankton, Pak Mun Reservoir | Lamkom and Kaewlurn 2001 |
|  | Rice paddy field with non-organic farming, Surin Province | Mungkung et al. 2014 |
| ***M. foliacea* var. *spinosa*** | | |
| **ASIA:** |  |  |
| Malaysia | Tasek Bera Wetland and forest swamp lake | Prowse 1969; Ratnasabapathy and Kumano 1974 |
