# Supplementary material for: ﻿Morphology, taxonomy, biogeography and ecology of Micrasteriasfoliacea Bailey ex Ralfs (Desmidiales, Zygnematophyceae)
Source: PhytoKeys. 2023 May 9;226:33–51. doi: 10.3897/phytokeys.226.103500 (PMC10189646; doi:10.3897/phytokeys.226.103500)
Supplement: Supplementary material 4 — Supplementary references [file phytokeys-226-033_article-103500__-s004.docx]

**Supplementary Table 3**. Geographical distribution of *M. foliacea v*ar. *ornata* throughout the world. Habitat types and locations are indicated, together with the reference stating its presence.

| **CONTINENT/ COUNTRY/ STATE** | **HABITAT TYPE/LOCATION** | **REFERENCES** |
| --- | --- | --- |
| **AFRICA:** |  |  |
| Cameroon | Méfou Reservoir | Atangana Étémé and Couté 1985 |
| Sudan | No details | Iltis 1980 |
| Swaziland | Pools, Usuto River | Williamson 1994 |
| Madagascar | Rapid water on gneiss, Ankazobé | Bourrelly and Couté 1991 |
| Mali | Alamba-Guindé, Middle Niger River | Couté and Rousselin 1975 |
| Niger | Niger River | Tahirou 2013 |
| Nigeria | Ikpoba Reservoir, Benin | Kadiri 2002 |
| **ASIA:** |  |  |
| China | Donghu Lake, Wuhan | Wei 1996 |
| Indonesia | Shallow rainwater pond, Ledo-Sanggau area, Borneo | Scott and Prescott 1961; Lenzenweger 1974 |
|  | Java and Sumatra | Scott and Prescott 1961 |
| Malaysia | Fresh waters | Prowse 1962 |
|  | Lake near Gerik, Tasek Bera Wetland | Williamson 1998 |
| Papua New Guinea | Lowland and medium altitude lakes and swamps | Vyverman 1991, 1992 |
| South Korea | Wetlands and oligotrophic rock pond, Jeju Island, Dongbaekdongsan | Kim 2013, 2014 |
| **AUSTRALIA:** |  |  |
| New South Wales | Glenbrook Lagoon, Blue Mountains | Atlas of Living Australia 2013 |
| Northern Territory | Mitchells Creek, near Darwin | Day et al. 1995; Ling and Taylor 2000; Skinner and Townsend 2005 |
| **NORTH AMERICA:** |  |  |
| Canada | Experimental Lakes Area, Northwest Territories | Findlay and Kling 1979 |
| USA: |  |  |
| Louisiana | Evangeline Parish, Lake Chicot | Prescott and Scott 1952; Prescott et al. 1977 |
| Florida | Dade-Collier Jetport, Dade County | Richardson 1973; Prescott et al. 1977 |
| **SOUTH AMERICA** |  |  |
| Argentina | Wetlands of Iberá, Lake Trin, Corrientes Province | Zalocar de Domitrovic 1981 |
| Brazil | Stagnant water, Lagoa Santa, Minas Gerais State | Nordstedt 1869 |
|  | Mato Grosso State | Borge 1925 |
|  | Santarém, Fazenda Taperinha, Pará State | Grönblad 1945 |
| Venezuela | Flooded savannah, Apure State | Salazár Pereira 1991; Salazar 2007‘2006’ |
